# Fibroblast-like synoviocytes exacerbate autoimmune arthritis upon psychological stress in an IL-6–dependent manner

**DOI:** 10.1126/sciadv.aec9257

**Published:** 2026-10-16

**Authors:** Jun Tamura, Shigeru Tanaka, Keishi Etori, Daisuke Hashiba, Shuhei Kameda, Atsuko Tomikawa, Ryo Kikuchi, Tatsuro Takahashi, Aiko Saku, Hirotoshi Kawashima, Junichi Nakamura, Seiji Ohtori, Shin-Ichiro Kagami, Koichi Hirose, Hiroshi Nakajima

**Affiliations:** ^1^ Department of Allergy and Clinical Immunology, Graduate School of Medicine, Chiba University, 1-8-1 Inohana, Chiba City, Chiba 260-8670, Japan.; ^2^ Department of Orthopaedic Surgery, Graduate School of Medicine, Chiba University, 1-8-1 Inohana, Chiba City, Chiba 260-8670, Japan.; ^3^ Department of Allergy and Rheumatology, International University of Health and Welfare Narita Hospital, 852 Hatakeda, Narita City, Chiba 286-0048, Japan.; ^4^ Research Center for Allergy and Clinical Immunology, Asahi General Hospital, 1326 I, Asahi City, Chiba 289-2511, Japan.

## Abstract

Psychological stress affects the immune system and exacerbates autoimmune diseases such as rheumatoid arthritis (RA), but the mechanisms underlying tissue inflammation during remission remain unclear. Here, we show that tube restraint stress exacerbates joint inflammation in a murine arthritis model during remission. This exacerbation required interleukin-6 (IL-6) produced by brown adipose tissue (BAT) following sympathetic activation. Fibroblast-like synoviocytes expressing the IL-6 receptor subunit gp130 were critical mediators, as IL-6 stimulated these cells to produce the neutrophil chemoattractant CXCL1. Pharmacological blockade of the β3-adrenergic receptor significantly attenuated stress-induced arthritis flares. In patients with RA, carriers of the hypofunctional Trp64Arg variant of *ADRB3* showed no positive correlation between psychological stress and disease activity, whereas noncarriers did. These findings identify a BAT–IL-6–fibroblast axis that reactivates inflammatory memory in arthritis and suggest that targeting stress-responsive pathways may provide a complementary therapeutic strategy alongside current RA treatments without broadly suppressing systemic immunity.

## INTRODUCTION

Psychological stress affects a variety of biological processes, including the immune system (*1*). It is well documented that acute psychological stress exacerbates inflammatory diseases (*2*). For instance, psychological stress can worsen bronchial asthma (*3*) and ulcerative colitis (*4*). The clinical course of rheumatoid arthritis (RA) is also affected by psychological stress (*5*–*7*). In patients with RA, increased serum levels of proinflammatory cytokines were observed during the experimental stress situations (*8*, *9*), indicating that the disease severity could be exacerbated by psychological stress through the cytokines; however, the underlying biological mechanisms are still largely unknown. One reason for the lack of progress on stress-induced exacerbation of RA is the absence of established animal models to evaluate arthritis deterioration caused by psychological stress. In addition, clinical studies that would involve imposing psychological stress on patients with RA to monitor disease exacerbation are ethically unacceptable, further limiting direct investigation in humans.

Psychological stress activates the hypothalamic-pituitary-adrenal axis and the sympathetic nervous system, leading to increased levels of glucocorticoids and catecholamines. While glucocorticoids generally exhibit immunosuppressive functions (*10*), they can also have proinflammatory effects under certain conditions (*11*, *12*), particularly during acute stress. Catecholamines bind to α-adrenergic receptors, activating mitogen-activated protein kinases (MAPKs), which promote the production of inflammatory cytokines in macrophages (*13*). Recent studies have also shown that catecholamines activate brown adipose tissue (BAT) via the β3 receptors, leading to the subsequent production of inflammatory cytokines, such as interleukin-6 (IL-6) (*14*–*17*). Consequently, acute psychological stress is a critical factor in initiating inflammatory responses. Patients with RA experiencing higher levels of psychological stress are more prone to disease flares (*7*, *18*). Conversely, inflammation affects depression and mood disorders (*19*, *20*). Thus, psychological stress and inflammation are closely interconnected, and understanding this relationship is crucial for elucidating the pathogenesis of immune-related diseases.

Although the prognosis of RA has markedly improved over the past two decades due to advances in disease-modifying antirheumatic drugs (DMARDs), the long-term use of DMARDs can lead to various health issues (*21*). The notable increase in severe infectious diseases is a major medical concern (*21*). By understanding and intervening in the pathways through which psychological stress worsens inflammatory diseases, a phenomenon commonly seen in modern societies, safer and more effective treatment strategies may be developed.

In the treatment of RA, controlling the recurrence of arthritis is a critical issue. Since synovitis directly leads to subsequent joint function deterioration (*22*), it is important to suppress arthritis recurrence as much as possible. It has been reported that arthritis recurrence tends to occur repeatedly in the same location (*23*), suggesting the presence of local inflammatory memory in the joints in addition to systemic immune memory such as autoantibodies. One possibility is that memory T cells remain in the synovium of the joints, potentially exacerbating inflammation in an antigen-dependent manner. Conversely, the involvement of pauci-immune mechanisms in refractory arthritis types has also been reported (*24*). Considering cases where arthritis worsens due to psychological stress, it is conceivable that an inflammation memory, stimulated independently of antigens, may exist; specifically, immune responses unrelated to memory T cells may be involved.

Here, we found that mice with antigen-induced arthritis (AIA) in a remission state experienced exacerbation of joint inflammation due to psychological stress. Our data indicated that psychological stress induced the production of IL-6 from BAT via β3-adrenergic receptor signaling, which subsequently stimulated fibroblast-like synoviocytes (FLS), leading to the recruitment of immune cells and the reactivation of synovial inflammation. An integrative analysis of *ADRB3* polymorphisms, psychological stress levels, and disease activity in patients with RA suggested that a similar stress-responsive mechanism may also be present in humans. Thus, our current study established an animal model to monitor the deterioration of arthritis triggered by psychological stress and shed light on the mechanism behind the exacerbation by psychological stress in patients with RA.

## RESULTS

### Tube restraint stress induces arthritis flares

To understand the mechanisms underlying stress-induced arthritis flares, we established an animal model. We used the AIA model, where joint inflammation could be triggered by a systemic antigen challenge in a remission state (*25*), as a positive control for assessing arthritis flares. Furthermore, this model allows for controlled localization of arthritis. This feature enables precise monitoring of subtle arthritis exacerbations by comparing joint thickness between affected and unaffected sides. We also used tube restraint as an acute psychological stress, where mice were kept in a 50-ml tube for 4 hours daily over 3 consecutive days (Fig. 1A) (*14*). In this model, corticosterone levels were significantly elevated during tube restraint stress, and the increase resolved within 4 hours (Fig. 1B). As previously described (*25*), methylated bovine serum albumin (mBSA)–immunized mice developed acute joint inflammation by the local injection of mBSA (Fig. 1C), and the acute joint inflammation was resolved within 3 weeks after the intra-articular mBSA injection (Fig. 1C). After the resolution of the joint inflammation, the systemic introduction of mBSA flared mild inflammation in the joint where the initial inflammation occurred (Fig. 1, C and D). The tube restraint flared the joint inflammation (Fig. 1, C and D). The ankle thickness in the tube restraint group increased over time and reached a similar degree to that in the mBSA challenge group (Fig. 1, C and D). We also analyzed the histopathological findings of the ankle joints. Consistent with the increased ankle thickness, the tube restraint group showed significantly higher histopathological scores than the untreated group (Fig. 1, E and F). To confirm that a similar arthritis flare was induced in knee joints by the tube restraint procedure, we performed histopathological analysis of the knee joints. Similar to the ankle joints, mice subjected to restraint stress exhibited greater synovial inflammation in the knee joints, although mild synovial proliferation was also observed in untreated samples (fig. S1, A and B). We also determined the levels of *Il6* in the synovium by quantitative polymerase chain reaction (qPCR) as an indicator of tissue inflammation and found that *Il6* was elevated in the tube restraint group (Fig. 1G). These results indicate that AIA is an appropriate mouse arthritis model for assessing the effects of psychological stress on joint inflammation flares, and acute psychological stress by the tube restraint procedure triggers measurable arthritis flares.

**Fig. 1.**
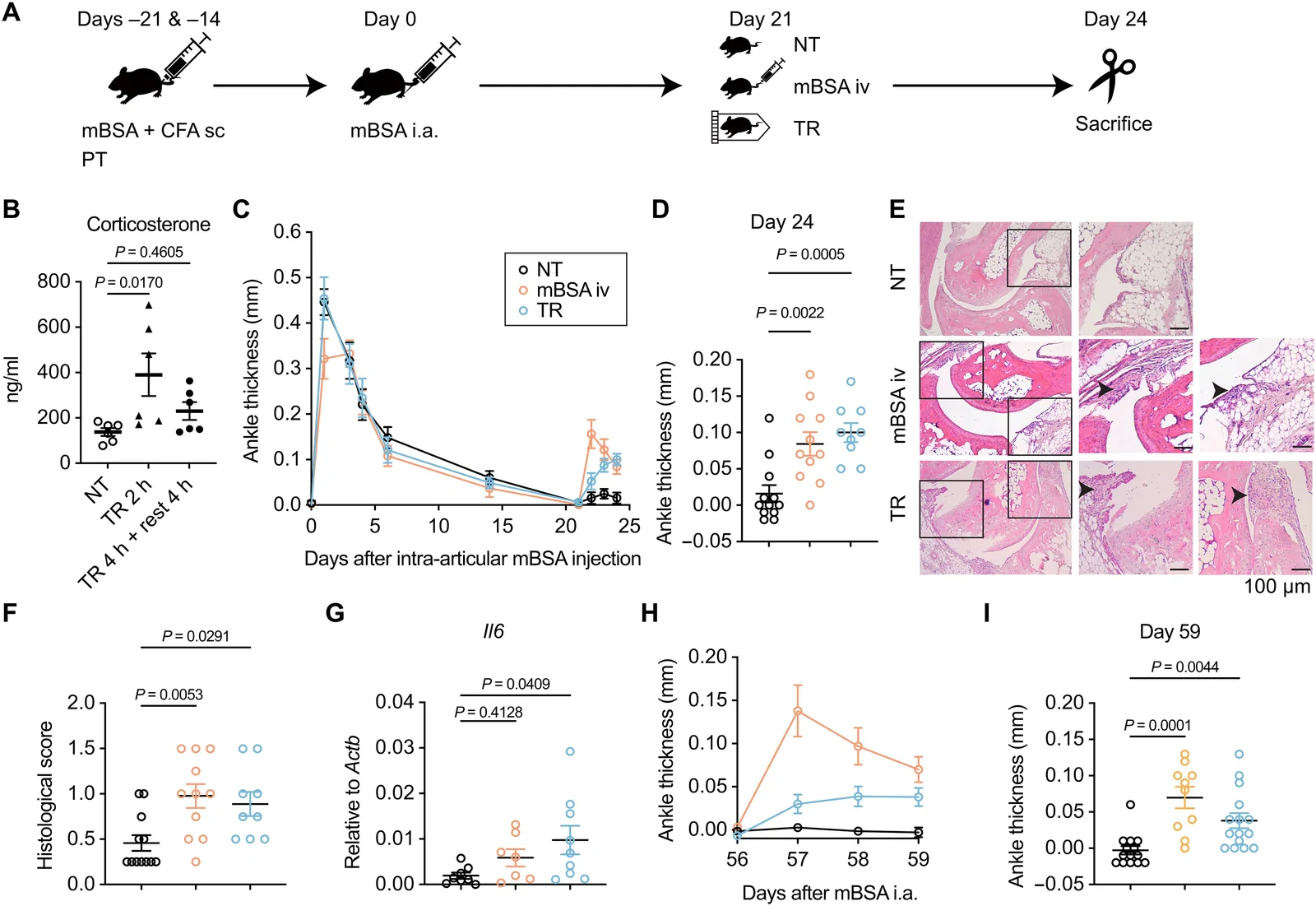
Tube restraint stress induces arthritis flares. (**A**) Three weeks after the mBSA intra-articular injection (i.a.), mice received either intervention, untreated (NT), mBSA intravenous (iv) injection, or tube restraint (TR). (**B**) Corticosterone levels after TR. *N* = 6 from two independent experiments. (**C**) Ankle thickness over time after the mBSA i.a. (**D**) Ankle thickness at day 24. (**E**) Representative histology of the ankle. Arrowheads indicate synovial inflammation. (**F**) Cumulative data of the histological score. (B to E) *N* = 9 to 12 from three independent experiments. (**G**) *Il6* expression in the synovial tissues determined by qPCR. *N* = 7 to 9 from two independent experiments. (**H**) Eight weeks after the mBSA i.a., mice received intervention, NT, mBSA intravenous, or TR. Ankle thickness after the intervention. (**I**) Ankle thickness at day 59. (H and I) *N* = 10 to 15 from four independent experiments. Statistical analyses were done by one-way analysis of variance (ANOVA) test followed by Dunnett test. sc, subcutaneous.

We performed a similar model in which mice were rested for 8 weeks after the local mBSA challenge to investigate whether arthritis flares up upon the tube restraint after prolonged remission. In this experimental setting, we still observed the joint inflammation in the tube restraint group, although the inflammation was relatively mild (Fig. 1, H and I). This suggests that the memory of the joint inflammation is maintained for at least 2 months.

### No obvious cartilage damage was detected in remission-phase AIA joints before stress exposure

Intra-articular injection of mBSA may cause cartilage damage. In addition, acute inflammation during the induction phase of AIA may also contribute to joint damage. To examine whether such joint damage mediates tube restraint–induced arthritis flare, we compared the extent of cartilage damage between naïve mice and mice with AIA in the remission phase. In most samples, Safranin O staining revealed no obvious cartilage damage in either naïve mice or mice with AIA in remission (fig. S1, C and D). These results suggest that cartilage damage is not required for tube restraint–induced arthritis flare.

### Tube restraint stress does not affect the antigen priming phase

We also examined whether the tube restraint affects the antigen-priming phase in AIA. In this experiment, mice were subjected to the tube restraint procedure before mBSA immunization (fig. S2A). Contrary to our expectations, the ankle thickness was comparable between the tube restraint and control groups (fig. S2B). Furthermore, neither the production of inflammatory cytokines nor the percentage of regulatory T cells showed significant differences (fig. S2C). These findings suggest that psychological stress does not influence disease severity during the antigen-priming phase in AIA.

### T and B cells are not required for arthritis flares due to tube restraint stress

It has been reported that the joint inflammation in AIA is mainly mediated by CD4^+^ T cells (*25*). A recent report also suggested the importance of CD8^+^ T cells in the recall phase in a modified AIA model (*26*). To address whether T cells are required for the arthritis flares caused by the tube restraint, isotype, anti-CD4, or anti-CD8 antibodies were administered to AIA mice before the tube restraint procedure (Fig. 2A). As anticipated, anti-CD4 and anti-CD8 antibodies completely depleted the corresponding T cells from the peripheral blood (fig. S3, A and B). Sixty to 80% of T cells in the synovium were CD4^+^ T cells, and CD8^+^ T cells were less common (fig. S3, C to F). CD4^+^ T cells and CD8^+^ T cells almost completely disappeared in the synovium in the anti-CD4 antibody-injected group in the knee joints (fig. S3, C and D). CD8^+^ T cells were not affected by anti-CD4 antibody treatment in the ankle joints (fig. S3, E and F), suggesting a possible difference in the local immune environment between the knees and ankle joints. The anti-CD8 antibody–treated group also had a significantly lower proportion of CD8^+^ T cells, whereas CD4^+^ T cells were unaffected (fig. S3, C to F). Under these conditions, mice injected with anti-CD4 or anti-CD8 antibodies still exhibited joint inflammation by the tube restraint, although the increase in ankle thickness was less prominent (Fig. 2, B to E).

**Fig. 2.**
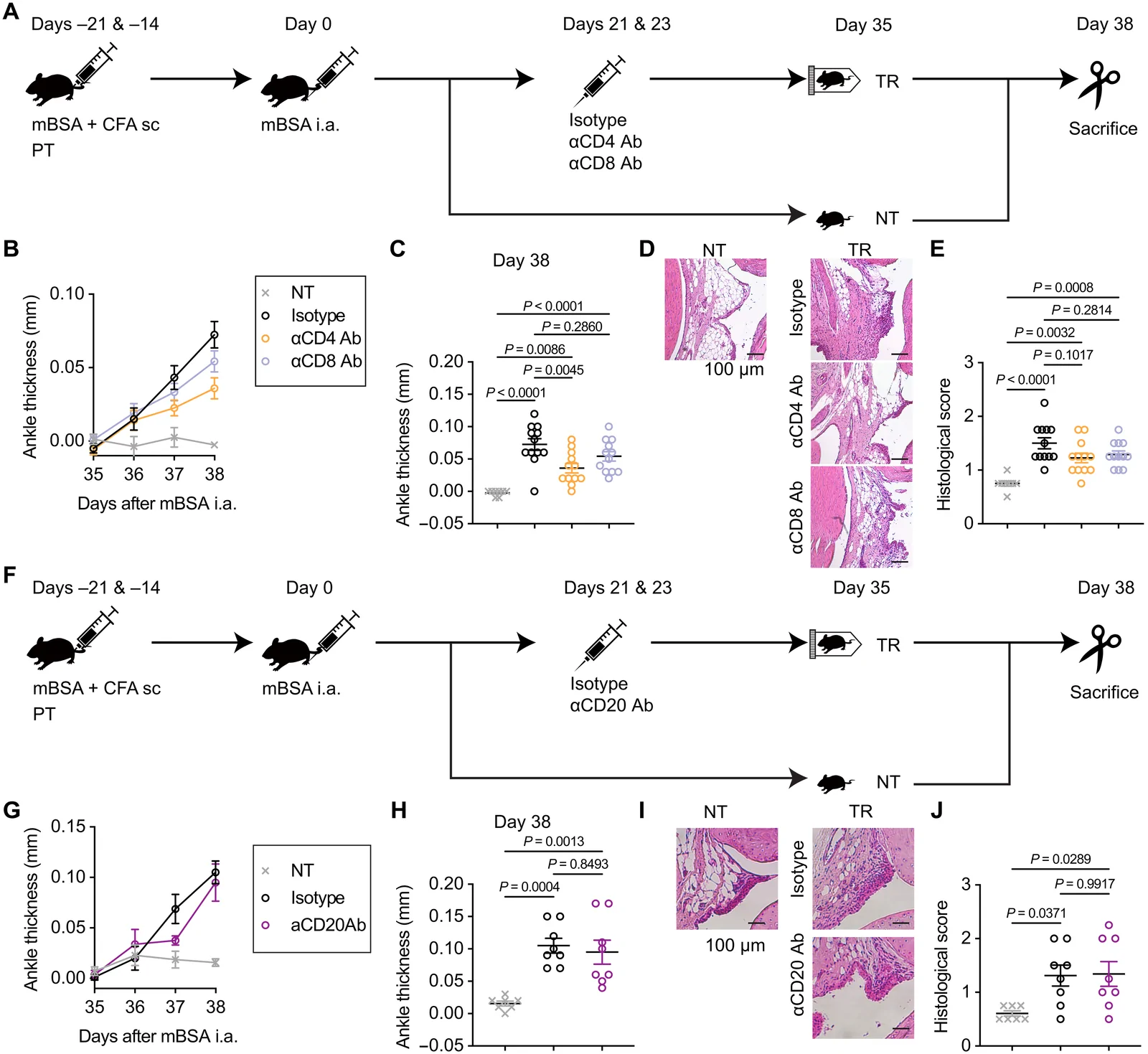
T and B cells are not required for arthritis flares due to tube restraint stress. (**A**) Three weeks after mBSA i.a., mice were treated with either an isotype control antibody, an anti-CD4 antibody, or an anti-CD8 antibody. The TR procedure was performed an additional 2 weeks later. (**B**) Ankle thickness after the intervention. (**C**) Ankle thickness at day 38. (**D**) The histology of the ankle. (**E**) Cumulative data of the histological score. (B and C) *N* = 8 to 12 from four independent experiments. Statistical analyses were done by one-way ANOVA test followed by Tukey test. (**F**) Mice were given either an isotype control antibody or an anti-CD20 antibody to deplete B cells before TR. (**G**) Ankle thickness after the intervention. (**H**) Ankle thickness at day 38. (**I**) The histology of the ankle. (**J**) Cumulative data of the histological score. (G to J) *N* = 7 to 8 from two independent experiments. Statistical analyses were done by one-way ANOVA test followed by Tukey test.

We also examined the role of B cells in stress-induced arthritis flare. To this end, mice were treated with either an isotype control antibody or an anti-CD20 antibody before the tube restraint (Fig. 2F). B cells were almost completely depleted from the synovium, although a small number of B cells remained in the peripheral blood (fig. S3, G to L). Under these conditions, B cell–depleted mice still exhibited arthritis flare (Fig. 2, G to J). Thus, neither T cells nor B cells are necessary for the stress-induced arthritis flares in AIA, but they may contribute to amplifying the inflammation.

### Sympathetic nerve-mediated BAT activation is required for the restraint stress–induced arthritis flares

A previous report demonstrated that acute psychological stress induces BAT activation through sympathetic nerve stimulation, which acts via β3-adrenergic receptors; this pathway is essential for triggering systemic inflammatory responses (*14*). Thus, we sought to determine whether this pathway is also vital for the arthritis flares caused by tube restraint. First, we assessed the effect of anesthesia, which blocks the consciousness of mice (fig. S4A). Vehicle-treated mice showed arthritis flares after the tube restraint as expected, while mice under anesthesia did not show increased ankle thickness (fig. S4, B to D), suggesting that normal consciousness is required for the arthritis flares. Next, chemical sympathetic nerve ablation was tested (fig. S4E). The administration of oxidopamine hydrobromide, a neurotoxic compound, reduced the increase in ankle thickness (fig. S4, F to H). Last, we removed BAT surgically before the tube restraint (fig. S4I). Again, surgical BAT removal dampened stress-induced joint inflammation (fig. S4, J to L). These results suggest that BAT activation by the sympathetic nerve system is vital for the stress-induced arthritis flares.

### IL-6 is responsible for arthritis flares due to tube restraint stress

It has been shown that acute psychological stress increases IL-6 levels, which are predominantly from BAT (*14*). Consistent with this, we observed enhanced IL-6 production in BAT after the restraint stress (fig. S5A). We also observed increased plasma IL-6 levels after the tube restraint procedure (fig. S5B). These findings led us to hypothesize that IL-6 is the corresponding cytokine that induces arthritis flares in response to psychological stress. To address this hypothesis, IL-6 pathway blockade therapy was tested. In this experiment, mice were given either an isotype control antibody or an anti–IL-6 receptor antibody every other day during the arthritis flare phase (Fig. 3A). As expected, the anti–IL-6 receptor antibody successfully reduced the increase in ankle thickness caused by the restraint stress (Fig. 3, B and C). Consistent with this, anti–IL-6 receptor antibodies also reduced the histopathological findings (Fig. 3, D and E).

**Fig. 3.**
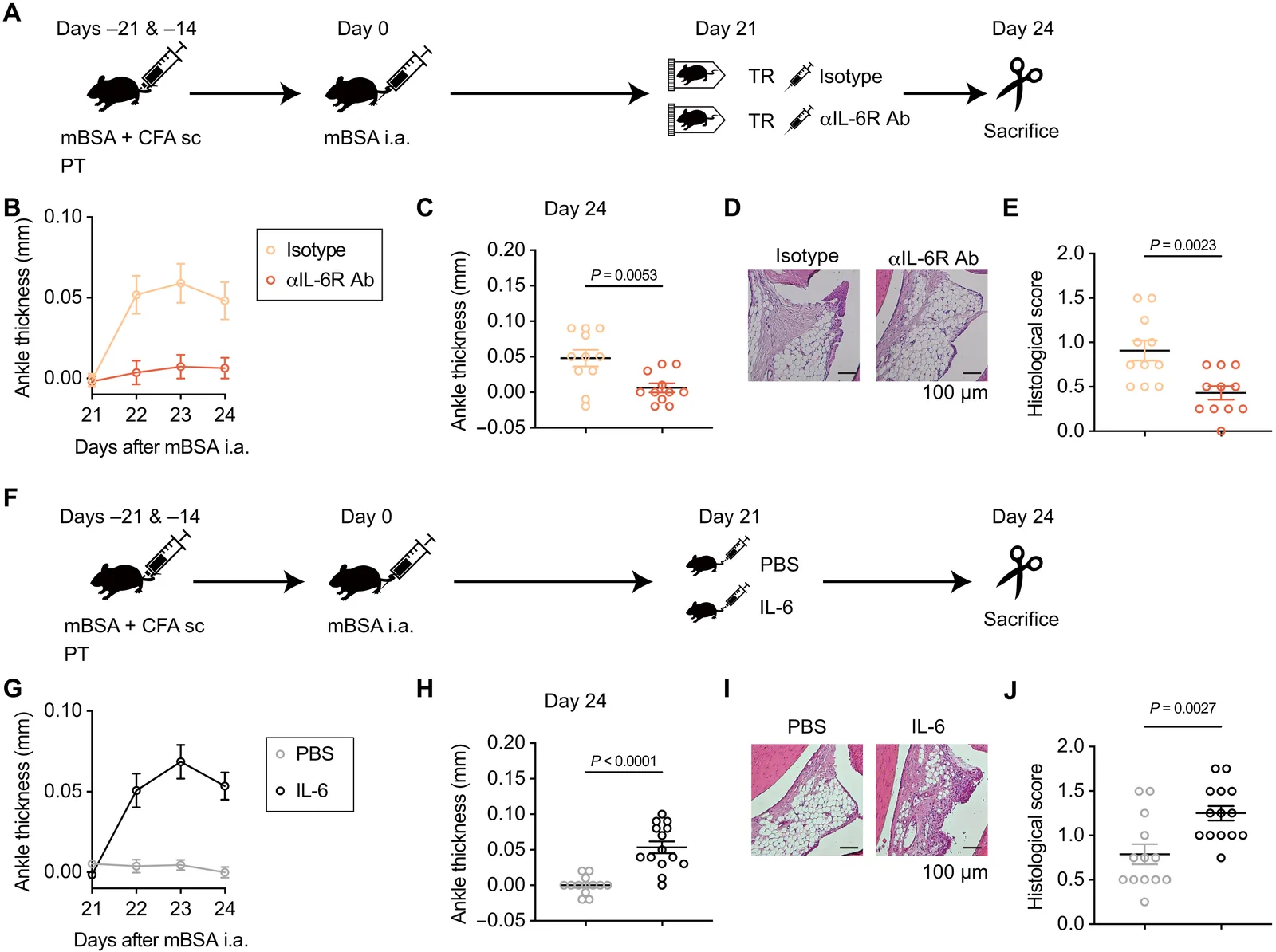
IL-6 is responsible for arthritis flares due to tube restraint stress. (**A**) The schematic protocol for anti–IL-6 treatment. (**B**) Ankle thickness after the intervention. (**C**) Ankle thickness at day 24. (**D**) Representative histology. (**E**) Cumulative data of the histological score. (B to E) *N* = 7 to 11 from four independent experiments. (**F**) The potential to flare arthritis by IL-6. The illustration shows how the mice were treated. (**G**) Ankle thickness after the intervention. (**H**) Ankle thickness at day 24. (**I**) Representative histology. (**J**) Cumulative data of the histological score. (G to J) *N* = 8 to 9 from two independent experiments. *P* values were determined by unpaired *t* test.

Then, we sought to determine whether IL-6 is sufficient to elicit the joint inflammation flares in AIA mice in remission. To test this, 2 ng of IL-6 was injected intravenously for 3 consecutive days in AIA mice that rested for 3 weeks after intra-articular antigen administration (Fig. 3F). IL-6 increased the thickness of the ankle joints, and the thickness was almost at the same levels as those in mice receiving the tube restraint procedure (Fig. 3, G and H). The histopathological scores were also significantly elevated in the IL-6–injected group (Fig. 3, I and J). Together, IL-6 is necessary and sufficient for eliciting the flares of preexisting arthritis in AIA.

### IL-6 receptor is dominantly expressed in FAP^+^ fibroblasts in the synovium

It is highly likely that IL-6 receptor–expressing cells in the synovium, a site of preexisting inflammation, respond to circulating IL-6 to promote joint inflammation flares. This hypothesis is based on the observations that (i) systemic IL-6 administration elicited the reaction, and (ii) no inflammation was observed outside the joints that had been locally injected with mBSA during the acute phase. To identify which cells in the synovial tissues express IL-6 receptors, in other words, the cells responsive to BAT-derived IL-6, we performed single-cell RNA sequencing (scRNA-seq). Synovial cells were harvested from the untreated mice, mice subjected to the tube restraint, and mice that received systemic mBSA injection, and these samples were subjected to scRNA-seq analysis. On the basis of the gene expression, cells were clustered into 11 different subsets (Fig. 4A and fig. S6A). Comparing the subset composition, both the tube restraint and systemic mBSA injection groups showed a higher proportion of Neutrophil2 that expressed several neutrophil activation markers such as *Ngp* and *Cd177*, suggesting the presence of active synovitis in these samples (Fig. 4B and fig. S6B). To our interest, Fibroblast2 was increased only in the tube restraint group (Fig. 4B). T cells constituted a less dominant population in our dataset (Fig. 4B). Next, we analyzed the differentially expressed genes in each cluster. Of note, *Il6st*, which encodes gp130, a component of the IL-6 receptor, was significantly up-regulated in Fibroblast2 (Fig. 4, C and D). *Fap* was also up-regulated in this subset, indicating that Fibroblast2 represents a proinflammatory synovial fibroblast population (Fig. 4, C and D) (*27*). We then sought to determine whether FAP^+^ fibroblasts express gp130 at a protein level. To address this, we performed flow cytometry (fig. S7). Consistent with the scRNA-seq findings, FAP^+^ fibroblasts from the synovial tissues of the untreated group expressed detectable levels of gp130 (Fig. 4, E and F). gp130 expression in FAP^+^ FLS was higher during the remission phase than in naïve mice, suggesting that arthritis induces gp130 expression in FLS (fig. S8). In addition to FAP^+^ fibroblasts, macrophages were also positive for gp130 (Fig. 4, E and F). We further investigated whether gp130 is expressed on human FLS in RA. For this, we analyzed synovial tissue from patients with RA and osteoarthritis (OA) by flow cytometry (fig. S9 and table S1). Although FLS from both RA and OA expressed detectable levels of gp130 (Fig. 4G), the expression intensity of gp130 was significantly higher in RA (Fig. 4H).

**Fig. 4.**
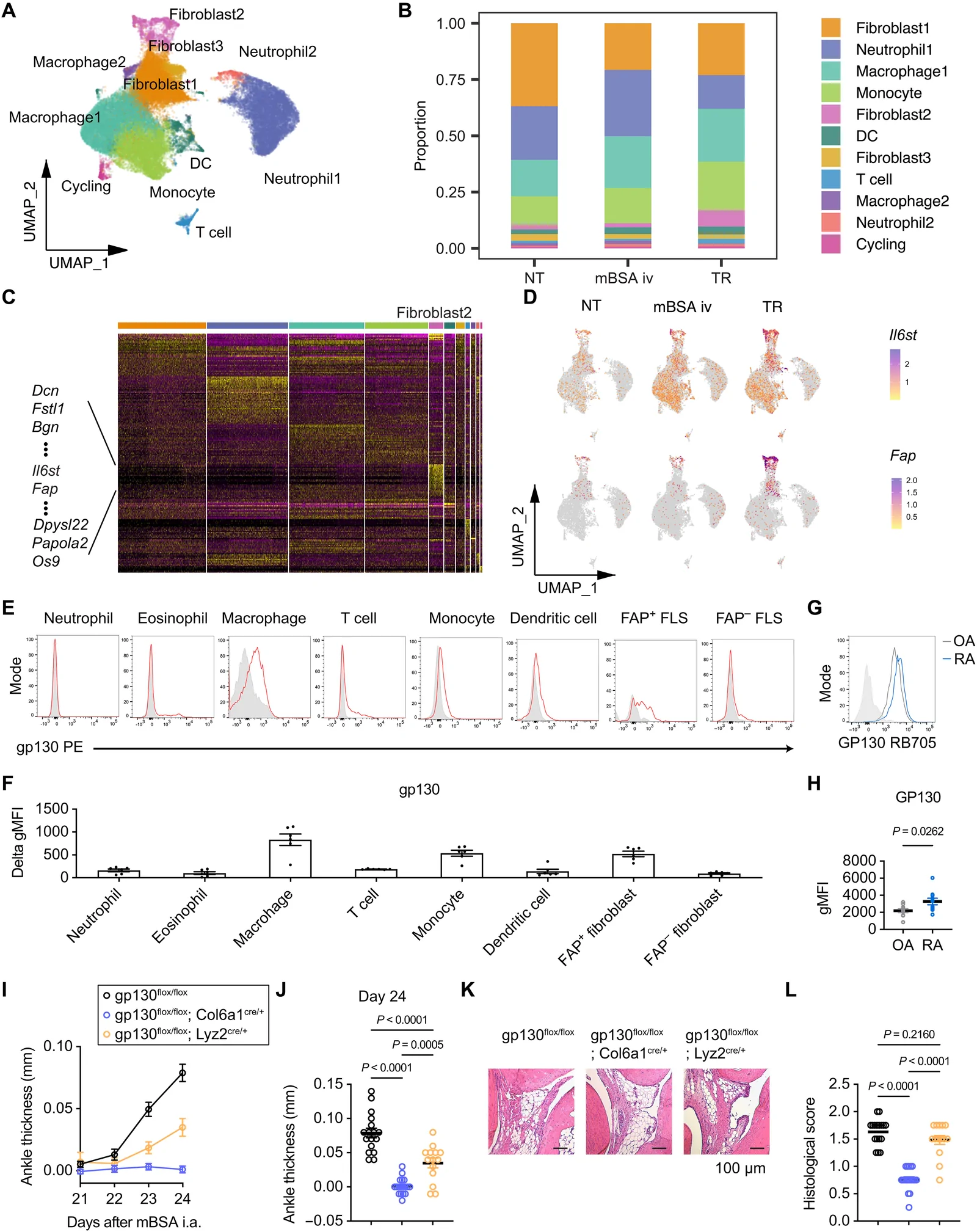
IL-6 receptor expression in FAP^+^ fibroblast is vital in stress-induced arthritis flare. (**A**) scRNA-seq of synovial cells in AIA mice. UMAP illustrating 11 clusters. (**B**) The proportion of clusters in each condition. (**C**) Differentially expressed genes (DEGs) in each cluster. Labeled gene names are DEGs in Fibroblast2 cluster. (**D**) The expressions of *Il6st* and *Fap* projected to UMAP. (**E**) Representative fluorescence-activated cell sorting (FACS) plots of gp130 expression in each synovial cell type under remission state. Gray indicates isotype control data. (**F**) Cumulative data of gp130 expression. The values represent each sample’s geometric mean fluorescence intensity (gMFI), normalized by subtracting the isotype control. *N* = 6 from two independent experiments. (**G**) Representative FACS plots of GP130 expression in FLS in RA and OA. (**H**) Cumulative data of GP130 expression in human FLS. *N* = 10 from two independent experiments. *P* values were determined by paired *t* test. (**I** to **L**) Fibroblast- or macrophage-specific conditional knockout of gp130 in the stress-induced AIA model. *N* = 14 to 18 from five independent experiments. (I) Ankle thickness after the intervention. (J) Ankle thickness at day 24. (K) Representative images of ankles. (L) Cumulative data of the histological score. *P* values were determined by one-way ANOVA test followed by Tukey test.

We then analyzed *Il6r*, which encodes IL-6Rα—another component of the IL-6 receptor—and found that neither FAP^+^ fibroblasts nor macrophages expressed IL-6Rα (fig. S10, A to C). IL-6 signaling occurs in gp130-expressing cells through two mechanisms: classical signaling via the membrane-bound IL-6Rα and trans-signaling via the soluble IL-6Rα (*28*). These results raised the possibility that FAP^+^ fibroblasts or macrophages respond to IL-6 via trans-signaling to promote joint inflammation.

### gp130 expression in FLS is essential for stress-induced arthritis flare

To further elucidate the cells responding to IL-6 within the synovium, we generated mice lacking gp130 in FLS (gp130^flox/flox^; Col6a1^cre/+^) or macrophages (gp130^flox/flox^; Lyz2^cre/+^) and performed the tube restraint–induced arthritis flare model (Fig. 4I). Col6a1^cre^-mediated deletion of gp130 was efficient (fig. S11A). In contrast, gp130 deletion in macrophages in gp130^flox/flox^; Lyz2^cre/+^ mice was incomplete; however, we observed a significant reduction in gp130 expression (fig. S11A). Ablation of gp130 in synovial fibroblasts increased the magnitude of acute inflammation induced by intra-articular mBSA injection through an unknown mechanism (fig. S11B). Despite this exacerbation of the initial inflammatory response, gp130^flox/flox^; Col6a1^cre/+^ mice showed no signs of the tube restraint–induced arthritis flare as measured by ankle thickness (Fig. 4, I and J). While ankle thickness was also decreased in gp130^flox/flox^; Lyz2^cre/+^ mice compared to controls, the reduction was more pronounced in gp130^flox/flox^; Col6a1^cre/+^ mice (Fig. 4, I and J). Consistent with these findings, the histopathological scores in gp130^flox/flox^; Col6a1^cre/+^ mice were significantly lower than in other groups (Fig. 4, K and L). These results suggest that IL-6 signaling in FLS is vital for the tube restraint stress–induced arthritis flare.

### FAP^+^ fibroblasts recruit immune cells through CXCL1

The recruitment of inflammatory cells is crucial for exacerbating tissue inflammation. To understand how synovial cells recruit inflammatory cells to the site upon the tube restraint, we performed a cell-cell communication analysis (*29*). Data from the tube restraint group were used in this analysis. There were extensive cell-cell communications among cells, with Fibroblast2 showing the most robust connections to other clusters (Fig. 5A). Pathway analysis was performed to understand the function of Fibroblast2. The most enriched pathway was the regulation of the apoptotic signaling pathway (Fig. 5B). Leukocyte migration and myeloid leukocyte migration were also enriched (Fig. 5B). Fibroblast2 had higher expressions of *Cxcl1* and *Cxcl12*, which are important for neutrophil and lymphocyte chemotaxis (Fig. 5C) (*30*, *31*). These findings indicate that FAP^+^ fibroblasts recruit immune cells through the production of chemokines.

**Fig. 5.**
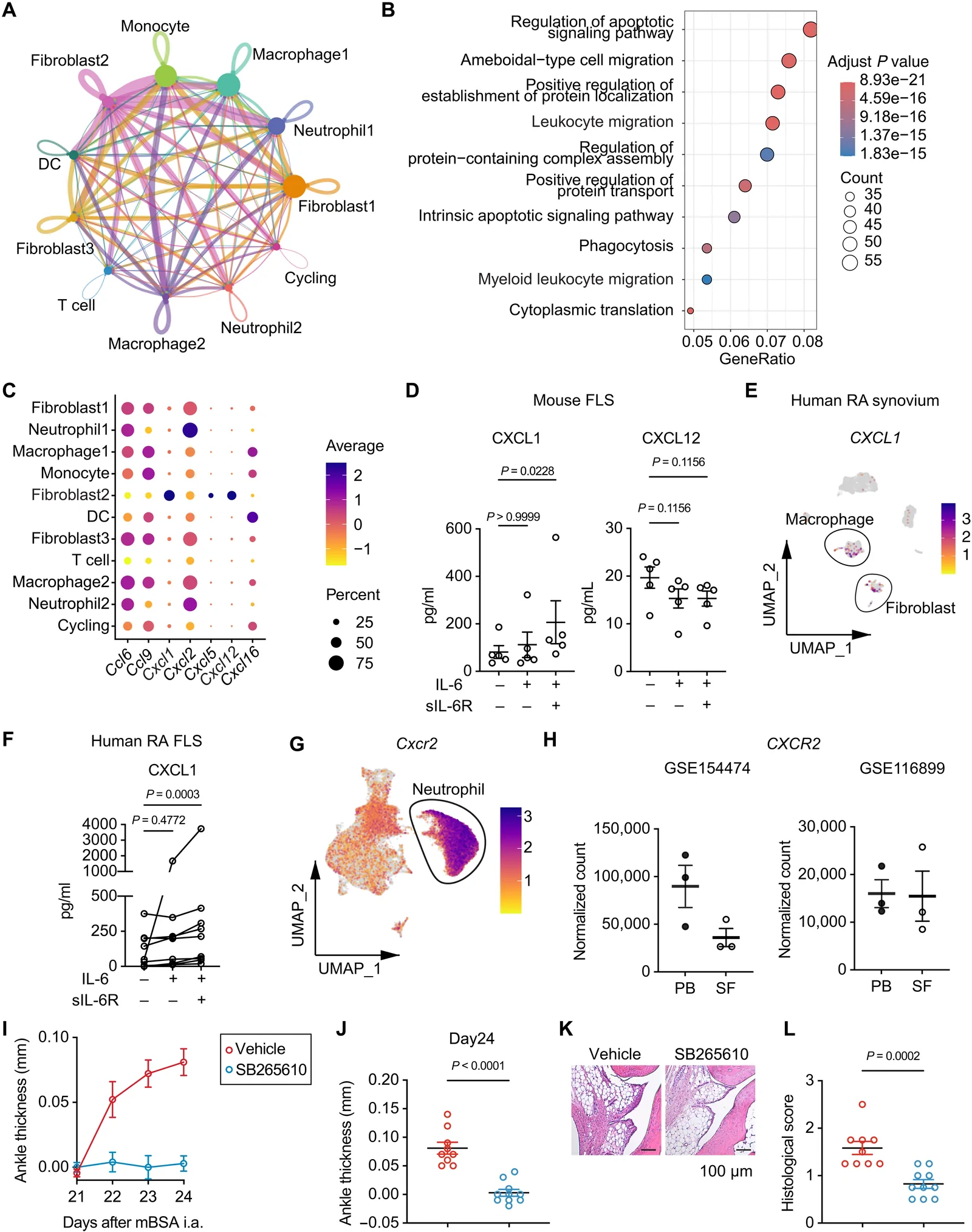
FLS recruit immune cells through CXCL1. (**A**) Cell-cell communication among clusters determined by CellChat. (**B**) GO term enrichment analysis in Fibroblast2. (**C**) Chemokine expression in each cluster. (**D**) CXCL1 and CXCL12 expression by IL-6/soluble IL-6Rα stimulation at protein levels in FLS in AIA. *N* = 5 from five independent experiments. *P* values were determined by Friedman test followed by Dunn’s test. (**E**) CXCR2 expression in human RA synovial samples (phs001457.v1.p1). (**F**) CXCL1 expression by IL-6/soluble IL-6Rα stimulation at protein levels in human RA FLS. *N* = 9 from four independent experiments. *P* values were determined by Friedman test followed by Dunn’s test. (**G**) The expression of chemokine receptor *Cxcr2* in AIA. (**H**) The expression of CXCR2 in peripheral blood (PB) and synovial fluid (SF) neutrophils in RA (GSE154474 and GSE116899). (**I** to **L**) The chemokine receptor antagonist experiment in the stress-induced AIA model. *N* = 9 to 10 from three independent experiments. (I) Ankle thickness after the intervention. (J) Ankle thickness at day 24. (K) Representative images of ankles. (L) Cumulative data of the histological score. *P* values were determined by unpaired *t* test.

To investigate whether IL-6 mediates the induction of these chemokines, we isolated FLS from inflamed knee joints and stimulated them in vitro. IL-6 alone had little effect on CXCL1 production; however, costimulation with IL-6 and soluble IL-6Rα markedly increased CXCL1 levels (Fig. 5D). In contrast, CXCL12 expression remained low under both conditions (Fig. 5D). Consistent with these findings, analysis of a public scRNA-seq dataset from RA synovial tissue confirmed that FLS express *CXCL1* (Fig. 5E). We further validated this in human cells, showing that IL-6 plus soluble IL-6Rα significantly up-regulated CXCL1 expression in FLS (Fig. 5F and table S2), as observed in murine FLS. Moreover, our scRNA-seq data confirmed the expression of the Cxcl1 receptor *Cxcr2* in Neutrophil1 and Neutrophil2 clusters (Fig. 5G). Public RNA-seq datasets of RA neutrophils also showed notable *CXCR2* expression in both peripheral blood and synovial fluid (Fig. 5H).

To determine whether this axis is functionally important for stress-induced arthritis flares, we administered a CXCR2 antagonist (SB265610). Treatment with SB265610 significantly reduced joint inflammation triggered by psychological stress (Fig. 5, I to L). Collectively, these findings suggest that CXCL1 production by IL-6–stimulated FLS may play a central role in neutrophil recruitment to the joint and in initiating inflammation during the remission phase in AIA mice.

### β3-Adrenergic receptor antagonist efficiently ameliorates stress-induced arthritis flare

From the perspective of developing RA treatments with minimal risk of infection, therapeutic strategies that do not directly suppress the immune system may offer an intriguing alternative. In the context of psychological stress, we hypothesized that reversible blockade of the β3-adrenergic receptor pathway could suppress IL-6 production from BAT, thereby preventing the recurrence of arthritis. To test this, we administered a β3-adrenergic receptor antagonist (SR59320A) before the application of tube restraint stress (Fig. 6A). SR59320 significantly decreased the levels of plasma IL-6 after tube restraint stress (Fig. 6B). As anticipated, SR59320A effectively suppressed joint swelling induced by tube restraint stress (Fig. 6, C and D). This finding was further supported by significantly improved histopathological scores due to SR59320A (Fig. 6, E and F).

**Fig. 6.**
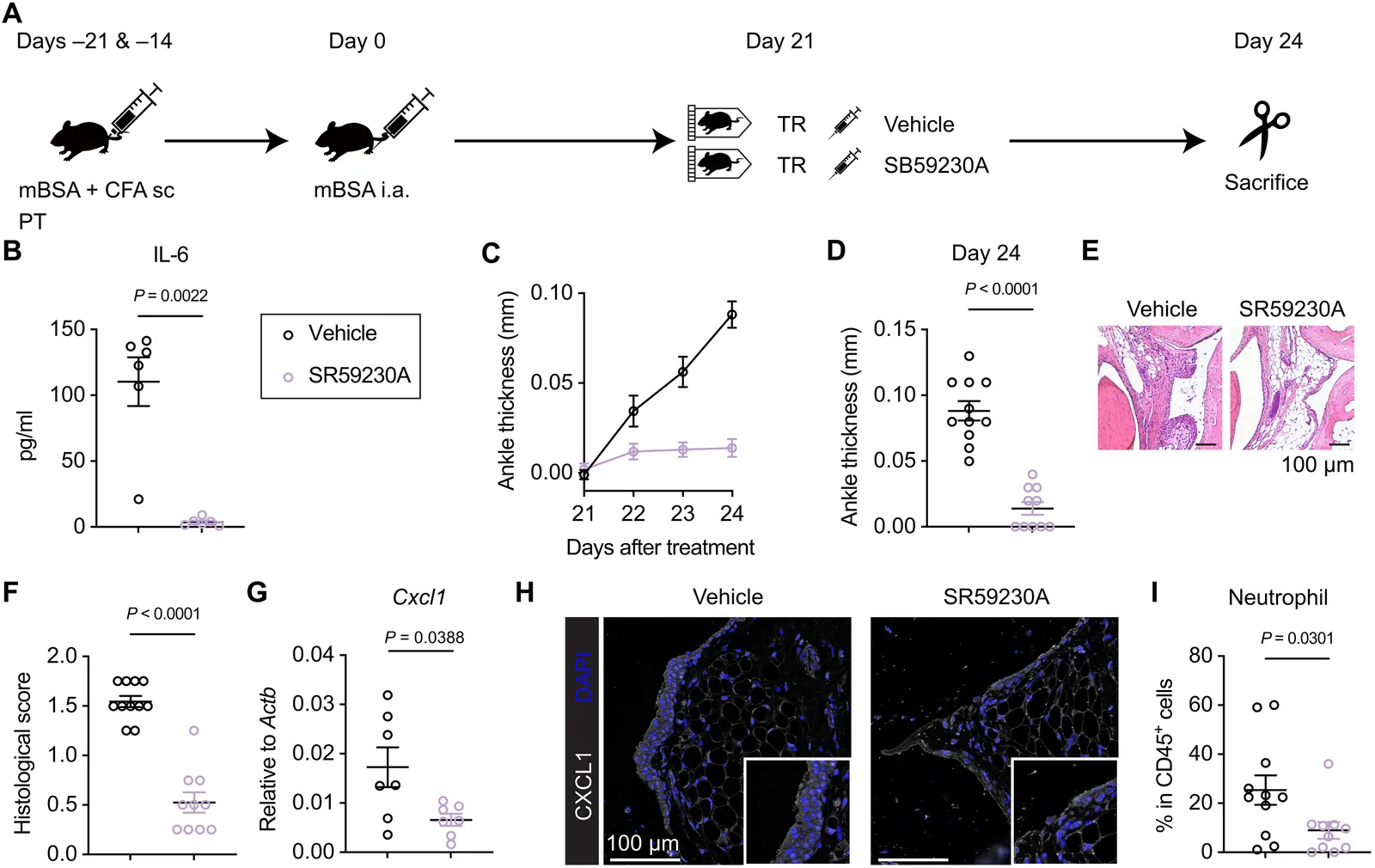
Successful treatment of stress-induced murine arthritis flare by a β3-adrenergic receptor antagonist. (**A**) Mice were administered either vehicle or SB59320A before the TR procedure. (**B**) Plasma IL-6 levels. *N* = 6 from two independent experiments. (**C**) Ankle thickness after the intervention. (**D**) Ankle thickness at day 24. (**E**) Representative images of ankles. (**F**) Cumulative data of the histological score. (C to F) *N* = 10 to 11 from four independent experiments. (**G**) *Cxcl1* expression in the synovial tissues. *N* = 7 from three independent experiments. (**H**) CXCL1 expression in the synovium by immunohistochemistry. Representative images are shown. DAPI, 4′,6-diamidino-2-phenylindole. (**I**) Neutrophil abundance in the synovial tissues. *N* = 10 to 11 from four independent experiments. *P* values were determined by unpaired *t* test.

We next examined whether the therapeutic interventions could attenuate the flare mechanism mediated by IL-6–activated FLS, specifically neutrophil recruitment via CXCL1. *Cxcl1* mRNA levels in the synovial tissue were significantly reduced by SR59320A (Fig. 6G). Immunostaining showed that CXCL1 protein was strongly expressed in hyperplastic lining cells and in cells within the sublining stroma (Fig. 6H). This expression was diminished following therapeutic intervention by SR59320A (Fig. 6H). Consistent with the reduction of CXCL1 in the synovium, the proportion of neutrophils was significantly decreased in mice treated with SR59320A (Fig. 6I and fig. S12). These findings demonstrate that targeting the β3-adrenergic receptor effectively suppresses stress-induced exacerbation of arthritis.

### IL-6 acts as a central upstream mediator of stress-induced arthritis exacerbation

Tumor necrosis factor (TNF) and IL-1β are known to directly stimulate FLS and induce IL-6 expression, thereby amplifying synovial inflammation through a feed-forward cytokine network (*32*, *33*). To evaluate the roles of this network in the stress–BAT activation–FLS activation axis, we measured plasma TNF and IL-1β levels after tube restraint stress. Although TNF levels were not altered, IL-1β levels were significantly elevated after the tube restraint (fig. S13A). We next treated AIA mice with either an isotype control antibody or an anti–IL-1β antibody to ask whether systemic IL-1β contributes to the reactivation of joint inflammation. We found that anti–IL-1β treatment efficiently reduced arthritis flare (fig. S13, B to E). However, up-regulation of *Cxcl1* in the synovium was still observed in anti–IL-1β (fig. S13F). These findings support a model in which IL-6 functions as a central upstream trigger of stress-induced arthritis flare, whereas IL-1β contributes to the amplification of inflammation.

### The functional *ADRB3* polymorphism affects the disease activity in patients with RA and psychological stress

To investigate the possibility that a stress-induced mechanism contributes to the exacerbation of arthritis in patients with RA, we focused on the presence of a functional polymorphism in the *ADRB3* gene, as direct induction of psychological stress in patients with RA is ethically and practically challenging. The Trp64Arg polymorphism of the *ADRB3* gene has been shown to confer reduced sensitivity to catecholamine stimulation (*34*). On the basis of this, we hypothesized that patients with RA carrying the Trp64Arg variant may exhibit attenuated IL-6 production from BAT in response to psychological stress, thereby preventing disease exacerbation by psychological stress. To test this hypothesis, we assessed the relationship between psychological stress and disease activity in patients with RA, stratified by *ADRB3* genotype. Consistent with previous reports (*35*), nearly one-third of patients carried at least one Trp64Arg mutant allele (table S3). Although the DMARDs used were different between the groups, the Clinical Disease Activity Index (CDAI), a composite measure of disease activity, and the Perceived Stress Scale (PSS) were comparable (table S3). In patients with only the wild-type allele, PSS levels showed a weak but significant positive correlation with the CDAI (Fig. 7). In contrast, this correlation was attenuated in patients carrying at least one low-sensitivity allele (Fig. 7). These findings suggest the presence of a mechanism in humans whereby psychological stress exacerbates arthritis via the β3-adrenergic receptor.

**Fig. 7.**
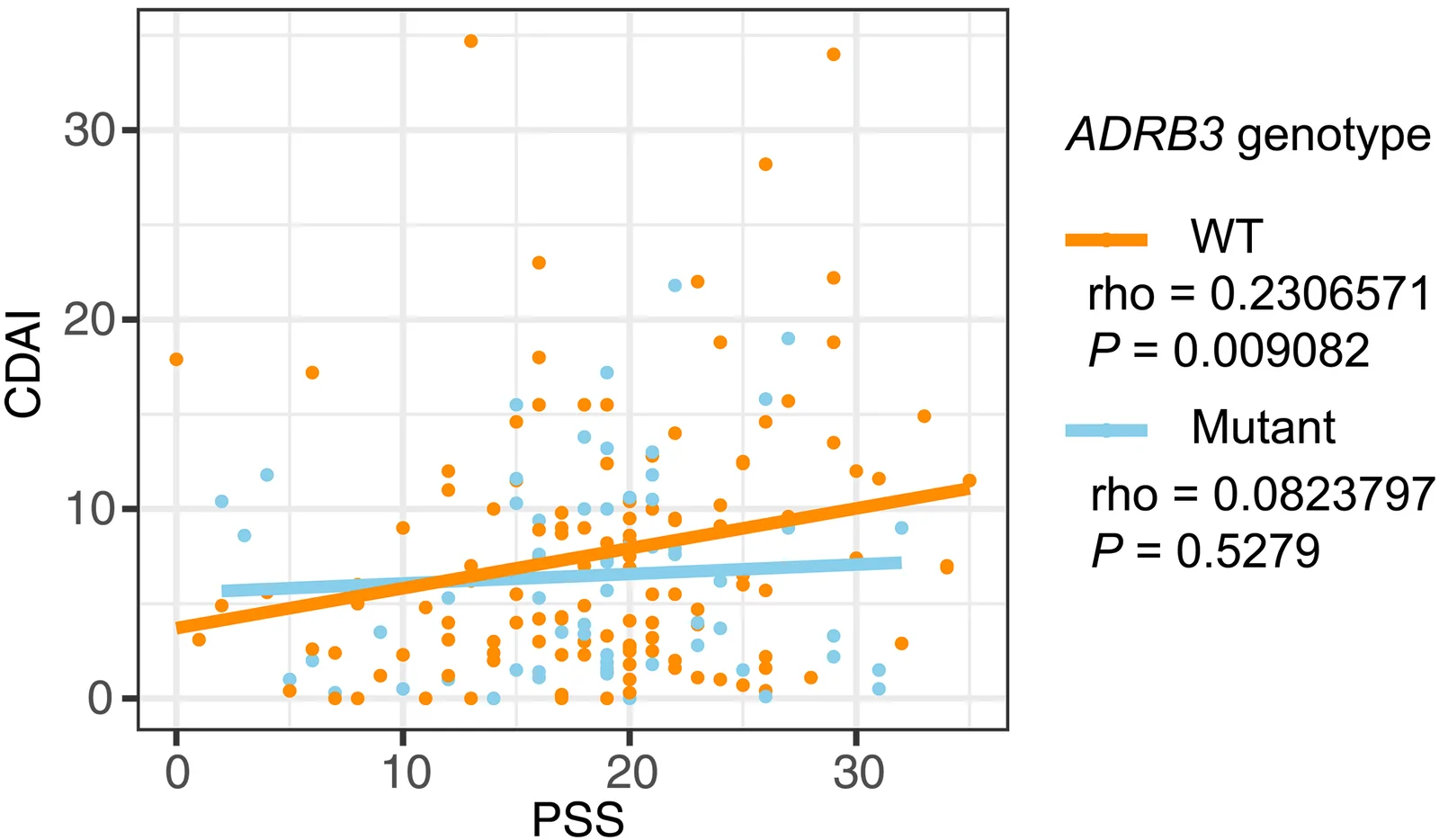
Correlation between RA disease activity and psychological stress is dependent on *ADRB3* genotype. Spearman’s rank correlation was performed between the CDAI and the PSS to assess the relationship in patients with RA stratified by a hypofunctional *ADRB3* variant. WT, wild type.

## DISCUSSION

The relationship between psychological stress and the exacerbation of RA has been recognized over the past few decades, but the underlying molecular mechanisms have remained unclear. This study found that psychological stress induced by the tube restraint stress reactivated AIA, a phenomenon similar to the stress-induced disease exacerbation observed in patients with RA. Although AIA is a T cell–dependent model, the stress-induced flares occurred even after T cell depletion, and the disease flared with IL-6 administration alone. These results suggest that IL-6 signaling in synovial non-T cells is crucial. FAP^+^ FLS expressed gp130, and FLS receiving IL-6 signaling were found to activate gene expression related to immune cell recruitment. We also found that CXCL1-mediated neutrophil recruitment played a crucial role. While detailed validation in humans is required, these findings hold the potential for developing a targeted therapeutic strategy.

Recent studies have revealed the importance of FLS in the pathogenesis of RA. Although FLS have been subdivided into two groups based on their histological distribution (*36*) (i.e., Thy1-negative lining and Thy1-positive sublining FLS), it has been reported that they exhibit a wide range of functions that this classification cannot fully explain (*28*, *37*, *38*). FAP expression was observed in both Thy1-negative lining and Thy1-positive sublining FLS in RA synovium. Only Thy1-positive sublining cells can exacerbate joint inflammation among FAP-positive FLS (*27*). In contrast, Thy1-negative lining FLS are strongly associated with bone erosion and exhibit a higher capacity for osteoclastogenesis (*27*). CXCL1 expression was observed in both the lining and sublining areas in the AIA synovium (Fig. 6H), suggesting that IL-6–responsive FLS may not be restricted by their distribution in the synovium.

It has long been believed that lymphocytes are the only cell type governing immune memory; however, recent studies have revealed that innate immune cells also have a memory of inflammation (*39*). This phenomenon is known as trained immunity, where innate immune cells that have encountered inflammation undergo epigenetic and metabolic reprogramming to elicit a rapid immune response upon subsequent stimuli. A recent report also suggests the presence of a similar phenomenon in synovial FLS (*40*). In this study, we observed the arthritis flares even after T and B cell depletion, suggesting that the memory of inflammation was not entirely dependent on those cells. In addition, the arthritis flares occurred in an antigen-independent manner, as IL-6 administration alone was sufficient to trigger the joint inflammation. IL-6R expression was observed in a few cell types, including FLS, and FLS may initiate inflammatory responses when they receive IL-6 produced during the tube restraint. These findings raise the possibility that FLS retain a persistent responsiveness during remission and that IL-6 may function as a trigger that recalls inflammatory responses. It remains unclear whether IL-6 is the only stimulant capable of triggering such responses in FLS. Although technically challenging, further investigation into the epigenetic and metabolic changes in FLS and the potential existence of a trained immunity–like mechanism is anticipated.

It is well established that IL-6 plays a critical role in the pathogenesis of RA (*41*). Antibodies targeting the IL-6R are widely used in RA treatment, and their clinical efficacy is remarkably high (*42*, *43*). In the RA joint, various cells, including macrophages and FLS, produce IL-6, creating an inflammatory network through their interactions. Our study demonstrated a phenomenon in which IL-6 originates from outside the joint and stimulates FLS, leading to the recurrence of arthritis. In our experimental model, stress-induced arthritis flare was suppressed by IL-6R blockade, supporting a central role for IL-6 signaling in this process. Notably, psychological stress can induce marked elevations in circulating IL-6 levels (*14*). Although species differences exist, such stress-induced increases in IL-6 may influence inflammatory activity even in patients receiving IL-6R blockade. Further clinical studies will be required to determine whether stress-induced IL-6 dynamics contribute to disease flares in patients treated with IL-6 pathway inhibitors.

Psychological stress has been reported to exacerbate disease severity in patients with RA (*7*, *18*, *44*). Our current research revealed that β3-adrenergic receptor blockade successfully suppresses stress-induced arthritis flares (Fig. 6). This finding suggests a potential therapeutic strategy targeting the sympathetic nervous system. The β3-adrenergic receptor is the primary receptor in BAT, the only tissue where β3-adrenergic receptors are predominant, aside from the bladder (*45*). It is also known that β3 receptors have less structural similarity to β1 and β2 receptors (*45*). Therefore, it may be possible to dampen BAT activation without affecting heart and lung function, where β1- and β2-adrenergic receptors are primarily distributed. It is also anticipated that this could lead to the establishment of therapeutic strategies for RA that do not directly influence antibacterial immunity. Further studies are required to develop this approach.

This study includes several limitations. First, stress-induced arthritis exacerbation was examined exclusively in the AIA model, and whether similar effects occur in other arthritis models remains to be determined. In addition, the 4-hour tube restraint procedure may induce proinflammatory responses other than those caused by psychological stress, such as mechanical injury or metabolic changes. Future studies using complementary stress models will be needed. Second, technical limitations should be considered in the analysis of synovial cells. Because murine synovial tissue is extremely small and dissociation efficiency may vary among samples, the recovered cells may not fully represent the intra-articular cellular composition. In addition, flow cytometric analysis of ankle synovium was technically challenging due to limited tissue availability, making direct cellular comparisons between ankle and knee joints difficult. Third, although we focused on CXCL1 as a downstream mediator of IL-6 signaling in FLS, the broader transcriptional and functional consequences of IL-6–signal transducer and activator of transcription 3 activation in FLS remain to be defined. The protective effect of β3-adrenergic receptor antagonism also requires further evaluation, including dose dependency, pharmacological specificity, and safety. Last, further evidence from human studies is necessary. Although we showed that IL-6 stimulation increases CXCL1 production in human FLS and that disease activity in stressed patients with RA may vary depending on β3-adrenergic receptor polymorphisms, whether psychological stress induces BAT-derived IL-6 in humans and thereby promotes CXCL1-dependent neutrophil recruitment in RA remains to be determined. In addition, the association between *ADRB3* polymorphisms and disease activity was based on an observational analysis and does not establish causality. Treatment differences between genotype groups and the use of the PSS, which captures subjective stress perception but not the full complexity or temporal dynamics of stress exposure, may also have influenced this association. Larger cohorts with detailed assessments of stress exposure and treatment variables will be needed to clarify the clinical relevance of this pathway to stress-associated RA exacerbation.

In conclusion, we developed a novel murine arthritis model that enables monitoring of disease flares triggered by psychological stress and found that FLS contribute to arthritis flares during remission in response to IL-6. This model is valuable for assessing stress-induced disease worsening in patients with RA and will facilitate the development of targeted therapeutic strategies to mitigate stress-induced RA flares.

## MATERIALS AND METHODS

### Study design

The aim of this study was to understand the underlying mechanisms by which psychological stress deteriorates or flares autoimmune arthritis. We developed a novel mouse model to dissect the molecular and cellular mechanisms of stress-induced arthritis deterioration. We performed several intervention experiments to demonstrate that psychological stress induces sympathetic nerve-mediated BAT activation and that the subsequent IL-6 release is vital in this model. We also identified the IL-6 responding cells in the synovium using scRNA-seq, flow cytometry, and fibroblast-specific gp130 knockout mice. IL-6–induced neutrophil chemoattractant levels in FLS were determined by enzyme-linked immunosorbent assay (ELISA). We applied a β3-adrenergic receptor antagonist for the treatment of stress-induced arthritis flare. Last, we assess the correlation between psychological stress and disease activity in patients with RA. This study was not blinded. Both male and female mice were used. Sample sizes were determined on the basis of prior experiments. The number of samples and independent experiments is indicated in the figure legends.

### Human subject

Patients with RA who fulfilled the 2010 ACR/EULAR criteria were recruited for the study (*46*). All participants were East Asians. Written informed consent was obtained from all participants in accordance with the Declaration of Helsinki. The study protocol was approved by the Chiba University Ethics Committee (approval numbers HS202106-06 and HS202501-01). The characteristics of the participants were summarized in tables S1 to S3. Psychological stress was assessed using the PSS (*47*).

### Mice

C57BL6/J mice were from CLEA Japan (Tokyo, Japan). Il6st^tm1Wme^ (B6 background), Tg(Col6a1-cre)1Gkl, and Lyz2^tm1(cre)Ifo^/J mice were described previously (*48*–*50*). Eight- to 12-week-old mice were used in this study. The mice were housed in specific pathogen–free facilities. The study was carried out in compliance with the ARRIVE guidelines. The Animal Care and Use Committee at Chiba University approved all protocols for animal experiments (approval number A8-008).

### Antigen-induced arthritis

AIA was induced as described previously (*51*). Briefly, mice were immunized at the flanks with 100 μl of mBSA (1 mg/ml) (Merck, Rahway, NJ) emulsified with complete Freund’s adjuvant [*Mycobacterium butyricum* (0.2 mg/ml)] (Merck). Mice were also given 200 ng of pertussis toxin intraperitoneally. A week after the initial immunization, the mice received the same immunization at the base of the tail. Two weeks after the second immunization, the right side of the ankle and knee joints was injected with 100 μg of mBSA in 5 μl of phosphate-buffered saline (PBS). The left side of the joints was injected with 5 μl of PBS as controls. To minimize animal usage, different joints were allocated for complementary analyses. The ankle joint was primarily used for clinical and histopathological evaluation, as joint swelling can be sensitively assessed in this region, whereas the knee joint was used for cellular and molecular analyses due to the relatively abundant synovial tissue that allows reliable isolation of stromal and immune cells. The ankle thickness was determined by measuring the difference between the left and right sides of the ankle using a digital caliper.

### Arthritis flares due to the tube restraint stress procedure

At least 3 weeks after the mBSA intra-articular injection, AIA mice were placed in a 50-ml tube for 4 hours each day for 3 consecutive days (*14*). In some experiments, 300 μg of mBSA was injected intravenously into some mice as a positive control group. The day after the final tube restraint procedure, mice were anesthetized and perfused with PBS for subsequent analyses, unless otherwise stated.

### Interventions and treatments for AIA mice

Anesthesia was administered using a mixture of anesthetic agents, including medetomidine (Meiji Animal Health, Kumamoto, Japan), midazolam (Nichi-Iko Pharmaceutical, Toyama, Japan), and butorphanol (Meiji Animal Health). Chemical sympathectomy was achieved with a 6-hydroxydopamine (150 mg/kg; Merck) intraperitoneal injection on the day before and the day of the start of the tube restraint procedure. BAT was surgically removed 2 weeks after the mBSA intra-articular injection, as previously described (*14*). For T cell depletion, 250 μg of rat immunoglobulin G2b (IgG2b) isotype control (BioXCell, Lebanon, NH), anti-CD4 antibody (GK1.5, BioXCell), or anti-CD8 antibody (2.43, BioXCell) was intraperitoneally injected twice at day 21 and day 23 after mBSA intra-articular injection. For B cell depletion, the same protocol was used except that either 250 μg of mouse IgG2c isotype control antibody (DV5-1, BioXCell) or anti-CD20 antibody (MB20-11, BioXCell) was intraperitoneally injected. Corresponding cell depletion was determined by flow cytometry 1 day after the second antibody injection. For IL-6 signal blocking, either 200 μg of rat IgG2b isotype control antibody or anti–IL-6R antibody (15A7, BioXCell) was intraperitoneally injected before the first and third tube restraint procedures. For TNF and IL-1β blocking, the same protocol was used, except that either 200 μg of polyclonal Armenian hamster IgG (BioXCell) or anti–IL-1β antibody (B122, BioXCell) was intraperitoneally injected. In Fig. 3F, 2 ng of recombinant mouse IL-6 (PeproTech, Cranbury, NJ) was intravenously injected for 3 consecutive days in place of the tube restraint to elicit arthritis flares. For the chemokine blocking experiment, SB265610 (2 mg/kg; Selleck, Houston, TX) or vehicle was injected intraperitoneally before the tube restraint procedure for 3 days. For the adrenergic receptor blocking experiment, SB59320A (10 mg/kg; Selleck) or vehicle was injected intraperitoneally before the tube restraint procedure for 3 days.

### Histopathology

Formalin-fixed paraffin-embedded (FFPE) hind paw samples were subjected to hematoxylin and eosin staining and Safranin O staining, and the inflammation scores were determined as previously described (*52*). Since we focused on assessing inflammation caused by the tube restraint stress, bone erosion and cartilage scores were not assessed in most experiments. The average histological scores of the talonavicular and talocrural joints were used for statistical analysis.

### Immunohistochemistry

For IL-6 staining, BAT FFPE samples were used. For antigen retrieval, slides were boiled in tris-EDTA (pH 9.0) antigen retrieval buffer (BioLegend, San Diego, CA) for 10 min. After antigen retrieval, slides were processed using the ImmPRESS HRP Universal PLUS Polymer Kit (Vector Laboratories, Newark, CA) according to the manufacturer’s protocol. Briefly, slides were blocked with 2.5% normal horse serum at room temperature for 30 min and then incubated with anti–IL-6 antibody (EPR23819-103, 1:100) (Abcam, Cambridge, UK) diluted in Can Get Signal Immunoreaction Enhancer Solution 1 (TOYOBO, Osaka, Japan) at room temperature for 1 hour. After washing, the slides were incubated with the secondary reagent for 30 min. IL-6 was then visualized using DAB solution. Representative images from three independent samples are shown for the nontreated, 2-hour restraint stress, and 4-hour restraint stress followed by 4 hours of rest groups.

Synovial tissue FFPE samples were used for CXCL1 staining, following a previously described protocol with some modifications (*53*). For antigen retrieval, slides were boiled in tris-EDTA (pH 9.0) antigen retrieval buffer for 10 min. After antigen retrieval, slides were blocked with 3% goat serum at room temperature for 30 min. Subsequently, slides were incubated overnight at 4°C with anti-CXCL1 polyclonal antibody (PA5-86508, Thermo Fisher Scientific, Waltham, MA), diluted 1:200 in Can Get Signal Immunoreaction Enhancer Solution 2 (TOYOBO). The next day, slides were washed and incubated with anti-Rabbit IgG Nano Recombinant Secondary Antibody conjugated with Alexa Fluor 647 (Thermo Fisher Scientific) for 2 hours at room temperature. Following washing steps, slides were counterstained with 4′,6-diamidino-2-phenylindole and visualized using an EVOS M7000 imaging system (Thermo Fisher Scientific).

### Synovial tissue digestion

Mouse synovial cells were isolated as previously described with some modifications (*54*). The skin and muscles were removed from the joint under a stereoscopic microscope. The hypertrophied synovial tissues were isolated. These specimens were sectioned into small pieces and subjected to vigorous shaking in a digestion buffer comprised of Liberase TL (25 μg/ml; Roche, Basel, Switzerland) and deoxyribonuclease I (DNase I; 1 mg/ml; Nippon Gene, Tokyo, Japan) in RPMI1640 medium (Merck) for 30 min at 37°C. The samples were subsequently filtered through a 50-μm cell strainer to obtain a single-cell suspension.

The cryopreserved human synovial tissues were digested as previously described (*55*). Briefly, thawed synovial tissue samples were mechanically disrupted using gentleMACS (Miltenyi Biotec, Deutschland, Germany) with the m_Spleen 04.01 setting. Disrupted samples were enzymatically dissociated in RPMI medium supplemented with Liberase TL (100 μg/ml) and DNase I (100 μg/ml) at 37°C for 30 min. Fragments were then passed through a 70-μm filter to obtain a single-cell suspension.

### Single-cell RNA sequencing

For the tube restraint group, mice were subjected to 4 hours of physical restraint per day for 3 consecutive days, and knee synovial tissue was collected the following day. For the mBSA group, mBSA was administered intravenously 1 day before knee synovial tissue collection. HIVE CLX scRNA-Seq Solution (Honeycomb biotechnologies, Waltham, MA) was used to collect single cells from synovial tissue, following the manufacturer’s protocol. Briefly, 30,000 cells were loaded into the HIVE collector for each condition. The HIVE collectors were kept at −80°C until use. Recovered cells were lysed, and mRNA was hybridized to poly(dT) beads. The beads were then collected, and first-strand cDNA was synthesized. Second-strand cDNA was generated using random primers, followed by amplification of the cDNA through 20 cycles of PCR. Indexes were added to the cDNA, and the products were purified using AMPure XP beads (Beckman Coulter, Brea, CA). The libraries were then sequenced on a DNBSEQ-G400RS (MGI, Shenzhen, China) in 100 base-paired-end mode. The obtained reads were mapped to the mm10 genome using BeeNet software (Honeycomb biotechnologies) and further analyzed using Seurat software (version 4.0) (*56*). Cells with genes ranging from 200 to 6000 were included in the analysis. Cells were also filtered on the basis of having less than 5% of mitochondrial genes. The expression matrix was normalized using Seruat’s SCTransform function. Three different batches were integrated using the IntegrateData command. We set PC30 as a cutoff for UMAP. We applied the FindNeighbors and FindClusters commands with a resolution of 0.3 to cluster cells. Differentially expressed genes were identified using the FindAllMarkers command, and adjusted *P* values of less than 0.05 were considered significant. GO enrichment analysis was performed using clusterProfiler (*57*). Cell-cell communication analysis was performed by CellChat (*29*). A similar procedure was applied to the deposit synovial cell data from patients with RA (dbGaP accession phs001457.v1.p1) (*58*), and *CXCL1* expression was projected onto the UMAP.

### Bulk RNA sequencing reanalysis

Publicly available RNA sequencing data (GSE154474 and GSE116899) were reanalyzed as previously described (*53*). Briefly, the deposit gene expression data were normalized using DESeq2 (*59*), and normalized *CXCR2* counts were presented as bar graphs.

### Flow cytometry

Cells were stained with Zombie NIR (BioLegend) to exclude dead cells from the analyses and subsequently incubated with purified anti-CD16/32 antibodies (BioLegend) to block Fc receptors. Then, samples were stained with antibodies in fluorescence-activated cell sorting (FACS) buffer for 30 min at 4°C. Cells were analyzed on BD Symphony A5SE (BD Biosciences). Cytokines and intracellular proteins were detected as previously described (*53*). Antibodies used for flow cytometric analyses in this study are listed in table S4.

### FLS culture

Mouse FLS were obtained as previously described (*60*) with some modifications. Briefly, the digested synovial sample from the knee joints was resuspended in Dulbecco’s minimum essential medium (DMEM), high glucose with GlutaMAX (Thermo Fisher Scientific) supplemented with 10% fetal bovine serum (Thermo Fisher Scientific), 1× nonessential amino acid (Thermo Fisher Scientific), 1 mM sodium pyruvate (Thermo Fisher Scientific), and penicillin/streptomycin (Thermo Fisher Scientific). The suspension was then seeded on a tissue culture plate coated with Cellmatrix Type I-C (Nitta Gelatin, Osaka, Japan). One hour after seeding, nonadherent cells were removed. The adherent cells were cultured and passaged at least two times, after which the expanded adherent cells were used. Cells were stimulated in the presence or absence of IL-6 (100 ng/ml) and recombinant IL-6Rα (100 ng/ml; R&D Systems, Minneapolis, MN) for 48 hours as indicated. Subsequently, the supernatants were collected and stored at −80°C until use for ELISA.

For human samples, digested synovial tissue was seeded in six-well plates and cultured in DMEM supplemented with 10% FBS. Two hours after seeding, nonadherent cells were removed, and the adherent cells were further cultured for at least 2 weeks. When the cells reached semiconfluence, they were detached and reseeded into larger culture dishes. The expanded adherent cells were used for the subsequent experiment. Cells were reseeded and stimulated for 48 hours in the presence or absence of IL-6 (100 ng/ml; BioLegend) and recombinant IL-6Rα (100 ng/ml; R&D Systems). Subsequently, the supernatants were collected and stored at −80°C until use for ELISA.

### Enzyme-linked immunosorbent assay

Mouse CXCL1 and CXCL12 levels of the culture supernatant were determined using a Mouse CXCL1 ELISA kit (Proteintech, Rosemont, IL) or a Mouse CXCL12 ELISA Kit (Proteintech). Human CXCL1 was determined using a Human CXCL1 ELISA kit (Proteintech). Corticosterone was measured using a Corticosterone ELISA kit (Enzo Life Sciences, Farmingdale, NY).

### MSD assay

Mouse IL-6, TNF, and IL-1β levels in the plasma were determined using a custom MSD V-plex assay kit (Meso Scale Diagnostics, Rockville, MD).

### Quantitative polymerase chain reaction

Total RNA was purified from synovial tissue using TRIzol reagent (Thermo Fisher Scientific) and a Direct-zol RNA MicroPrep Kit (Zymo Research, Irvine, CA). Reverse transcription and qPCR were performed as previously described (*61*). The following PCR primers were used: *Il6* forward, 5′-ATCCAGTTGCCTTCTTGGGACTGA-3′; *Il6* reverse, 5′-TAAGCCTCCGACTTGTGAAGTGGT-3′; *Cxcl1* forward, 5′-TCCAGAGCTTGAAGGTGTTGCC-3′; *Clcl1* reverse, 5′-AACCAAGGGAGCTTCAGGGTCA−3′; *Actb* forward, 5′-GCTCTGGCTCCTAGCACCAT-3′; *Actb* reverse, 5′-GCCACCGATCCACACAGAGT-3′. The levels of target genes were normalized to the levels of *Actb*.

### Genotyping of the *ADRB3* polymorphism

Genomic DNA was extracted from peripheral blood samples by NucleoSpin Blood QuickPure (Takara) according to the manufacturer’s protocol. The *ADRB3* rs4994 polymorphism was detected using a real-time TaqMan allelic discrimination assay (C___2215549_20, Thermo Fisher Scientific).

### Statistics

Data are represented as means ± SEM. An unpaired *t* test, paired *t* test, one-way analysis of variance (ANOVA) test, or Friedman test was used to analyze unpaired normal distribution, paired data, parametric multivariate data, and nonparametric multivariate data, respectively. For the patient characteristics, either Wilcoxon rank sum test or Fisher’s exact test was used. The relationship between CDAI and PSS was assessed using Spearman’s rank correlation. Prism 10 (GraphPad Software, San Diego, CA) or R (version 4.4.3) was used for the analyses. Statistical significance was set at *P* < 0.05.
