# Supplementary material for: Fibroblast-like synoviocytes exacerbate autoimmune arthritis upon psychological stress in an IL-6–dependent manner
Source: Sci Adv. 2026 Oct 16;12(42):eaec9257. doi: 10.1126/sciadv.aec9257 (PMC13674482; doi:10.1126/sciadv.aec9257)
Supplement: Supplementary file 1 [file sciadv.aec9257_sm.pdf]

Science Advances
AAAS

Supplementary Materials for

Fibroblast-like synoviocytes exacerbate autoimmune arthritis upon psychological stress in an IL-6–dependent manner

Jun Tamura *et al.*

Corresponding author: Shigeru Tanaka, stanaka@chiba-u.jp; Hiroshi Nakajima, nakajimh@faculty.chiba-u.jp

Sci. Adv. **12**, eaec9257 (2026)
DOI: 10.1126/sciadv.aec9257

The PDF file includes:

Figs. S1 to S12
Tables S1 to S4
Legend for data file S1

Other Supplementary Material for this manuscript includes the following:

Data file S1

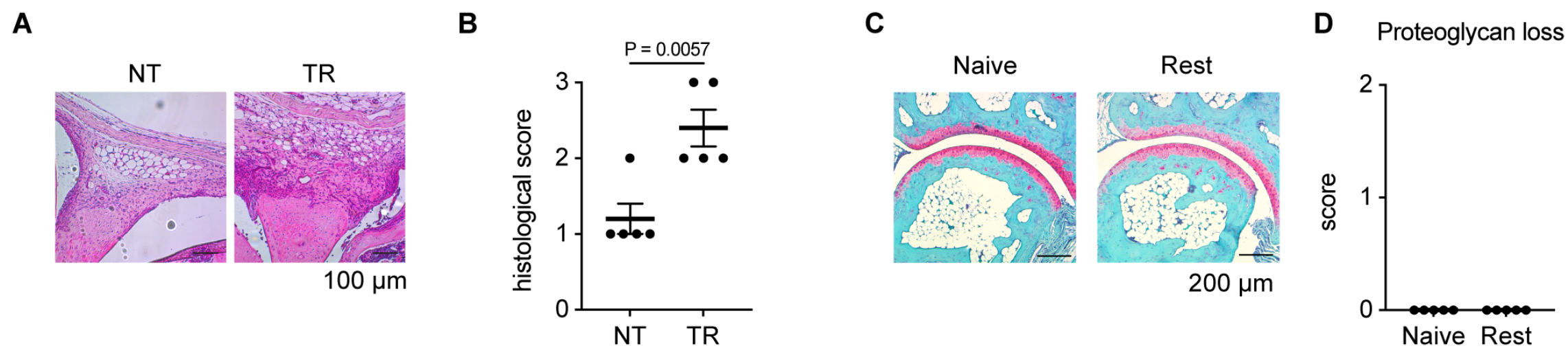

fig. S1. Histological assessment of knee joints and structural damage in ankle joints in mice subjected to tube restraint stress (A) Representative H&E-stained sections of knee joints from mice subjected to tube restraint stress (TR) or no treatment (NT). **(B)** Histological score of knee joints. N = 5 from 2 independent experiments. **(C)** Representative Safranin O-stained sections of ankle joints from naïve mice or AIA-induced mice after 3 weeks of rest. **(D)** Proteoglycan loss scores in ankle joints. N = 5 from 2 independent experiments. P values were determined by unpaired t-test.

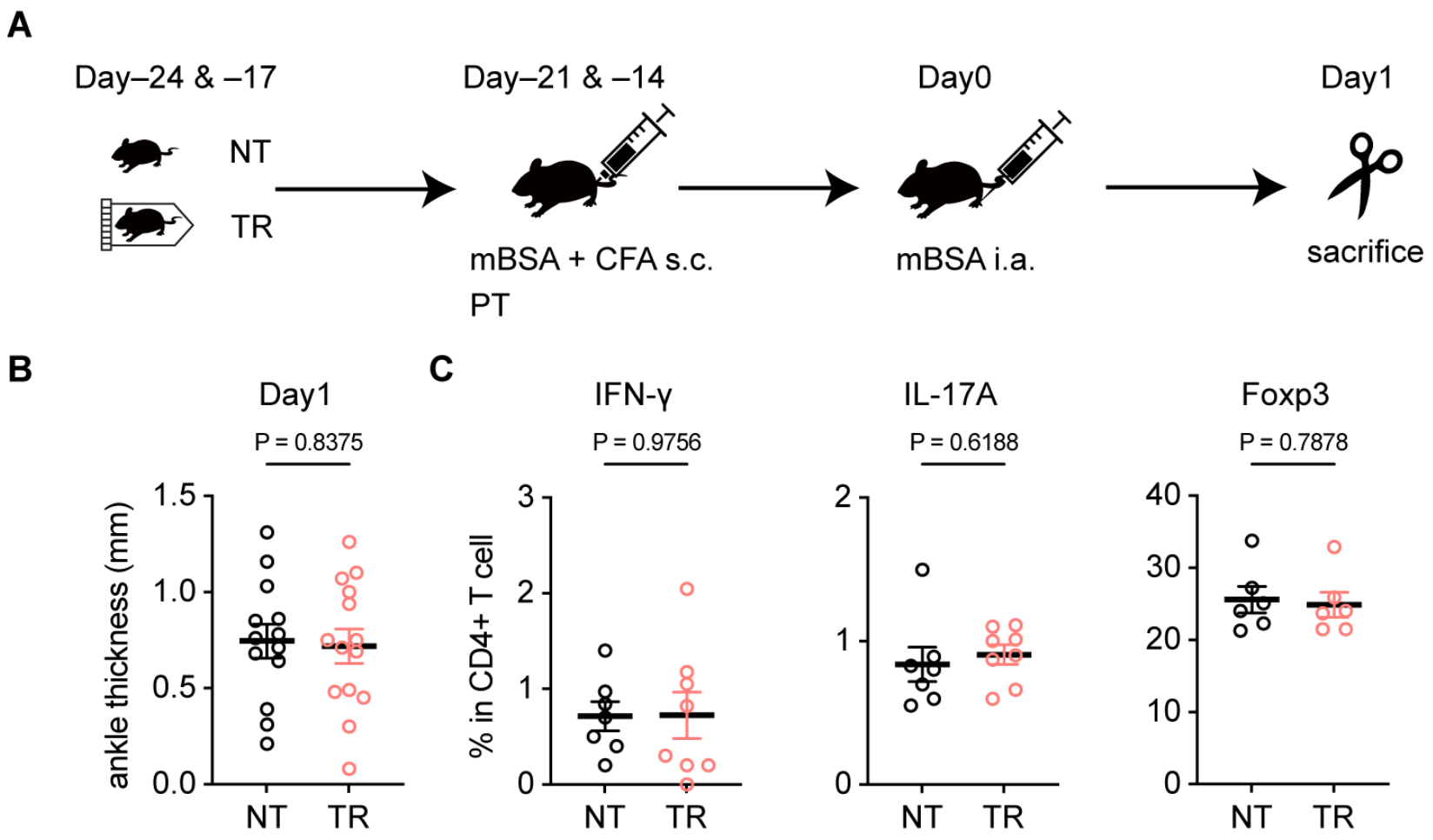

fig. S2. Tube restraint stress had no effect on the severity of AIA during the induction phase. (**A**) Mice were subjected to either tube restraint stress (TR) or no treatment (NT) for three consecutive days prior to immunization. N = 13 – 14 from 4 independent experiments. (**B**) Ankle thickness was measured at Day 1. (**C**) IFN-γ, IL-17A, or Foxp3-expressing CD4+ T cells in the synovial tissues. N = 6 – 8 from 2 independent experiments. P values were determined by unpaired t-test.

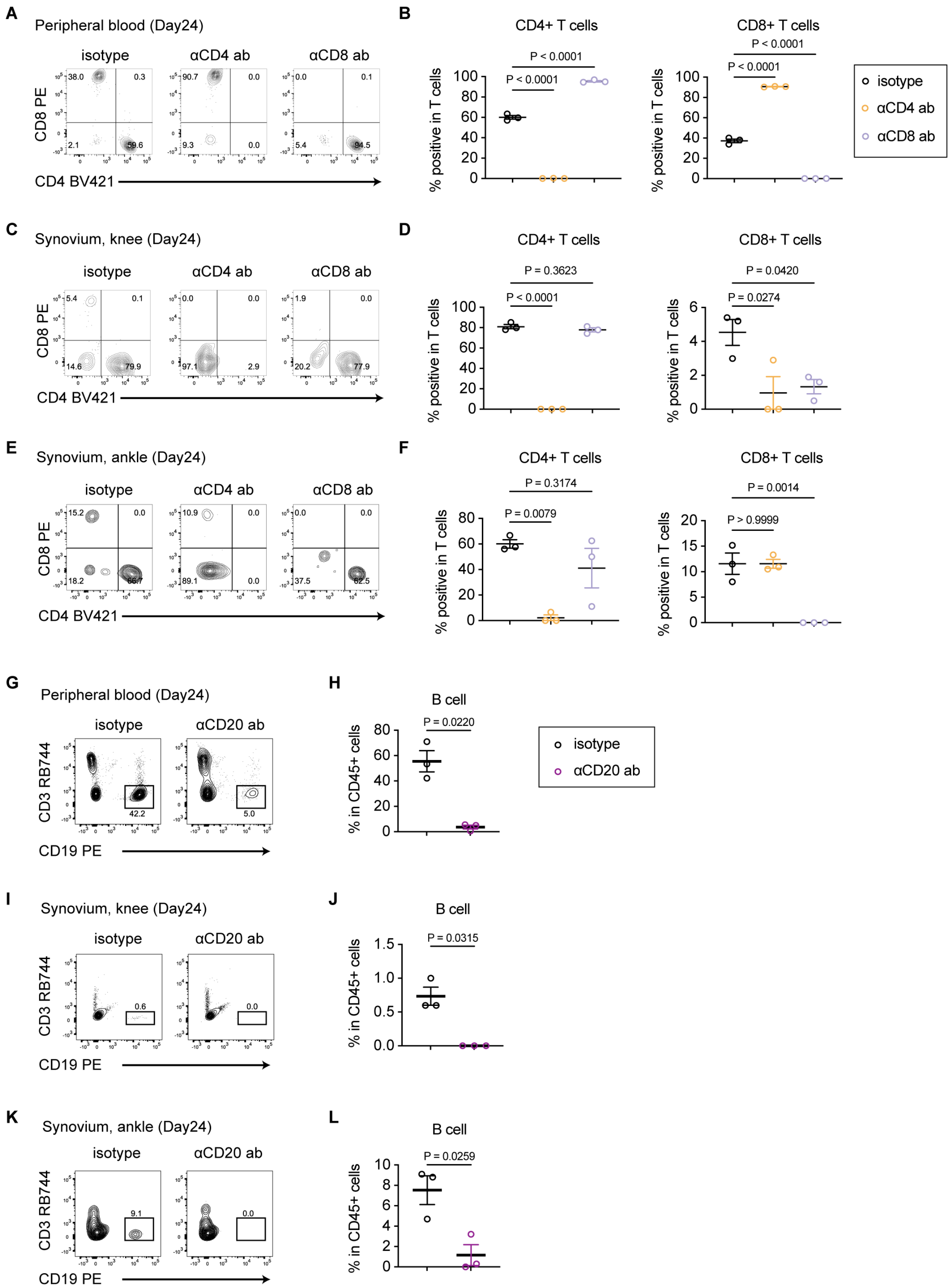

fig. S3. Depletion of T and B cells by specific antibodies. (A-F) Three weeks after intra-articular mBSA injection, mice were administered an isotype control antibody, an anti-CD4 antibody, or an anti-CD8 antibody. **(A)** Representative FACS plots of T cells in peripheral blood. Cells were gated on live CD45+ CD3+ cells. **(B)** Cumulative data of CD4+ and CD8+ T cells in peripheral blood. **(C)** T cells in knee joints. **(D)** Cumulative data of CD4+ and

CD8+ T cells in knee joins. **(E)** T cells in ankle joints. **(D)** Cumulative data of CD4+ and CD8+ T cells in ankle joins. **(G-L)** Three weeks after mBSA i.a., mice were administered an isotype control antibody or an anti-CD20 antibody. **(G)** Representative FACS plots of B cells in peripheral blood. Cells were gated on live CD45+ cells. **(H)** Cumulative data of CD19+ B cells in peripheral blood. **(I)** B cells in knee joints. **(J)** Cumulative data of CD19+ B cells in knee joins. **(K)** B cells in ankle joints. **(L)** Cumulative data of CD19+ B cells in ankle joins. N = 3. Statistical analyses were done by One-way ANOVA test followed by Dunnett test.

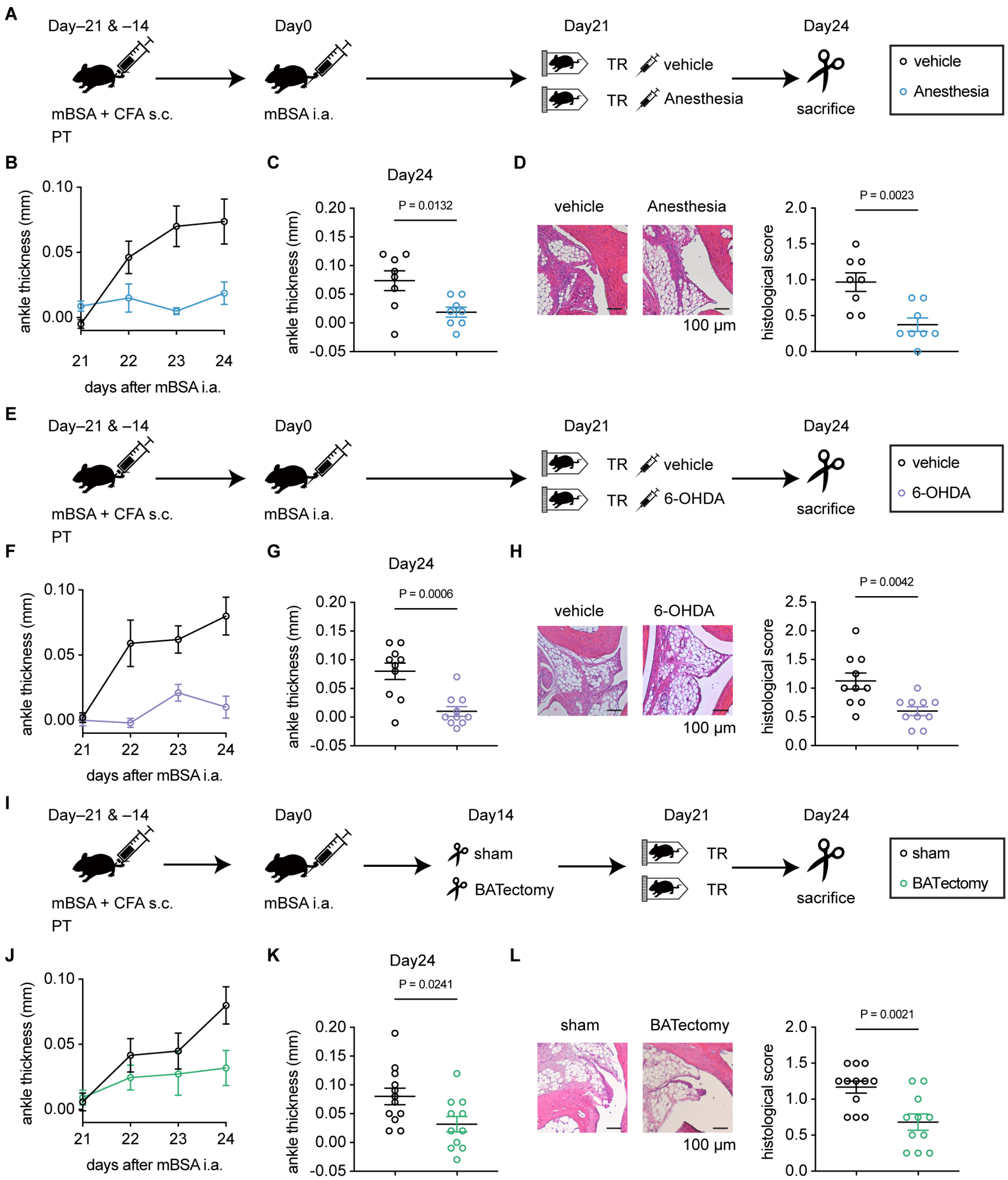

fig. S4. Sympathetic nerve - brown adipose tissue system is crucial for tube restraint-induced arthritis flares. (A) AIA-induced mice in remission were subjected to tube restraint (TR). Mice received either vehicle or intraperitoneal anesthetic agents during TR. **(B)** Ankle thickness after the intervention. (**C**) Cumulative data on ankle thickness at Day24. (**D**) Representative histology images and cumulative histological score. N = 7 – 8 from 3 independent experiments. (**E**) AIA-induced mice in remission were subjected to TR and received either vehicle or 6-OHDA. (**F**) Ankle thickness after the intervention. (**G**) Cumulative data on ankle thickness at Day24. (**H**) Representative histology images and cumulative histological score. N = 6 – 8 from 3 independent experiments. (**I**). AIA-induced mice in remission underwent either surgical brown adipose tissue excision (BATectomy) or a

sham operation. One week after the surgical procedure, mice were subjected to TR. (**J**) Ankle thickness after the intervention. (**K**) Cumulative data on ankle thickness at Day24. (**L)** Representative histology images and cumulative histological score. N = 11 – 12 from 5 independent experiments. Statistical analyses were done by One-way ANOVA test followed by Dunnett test.

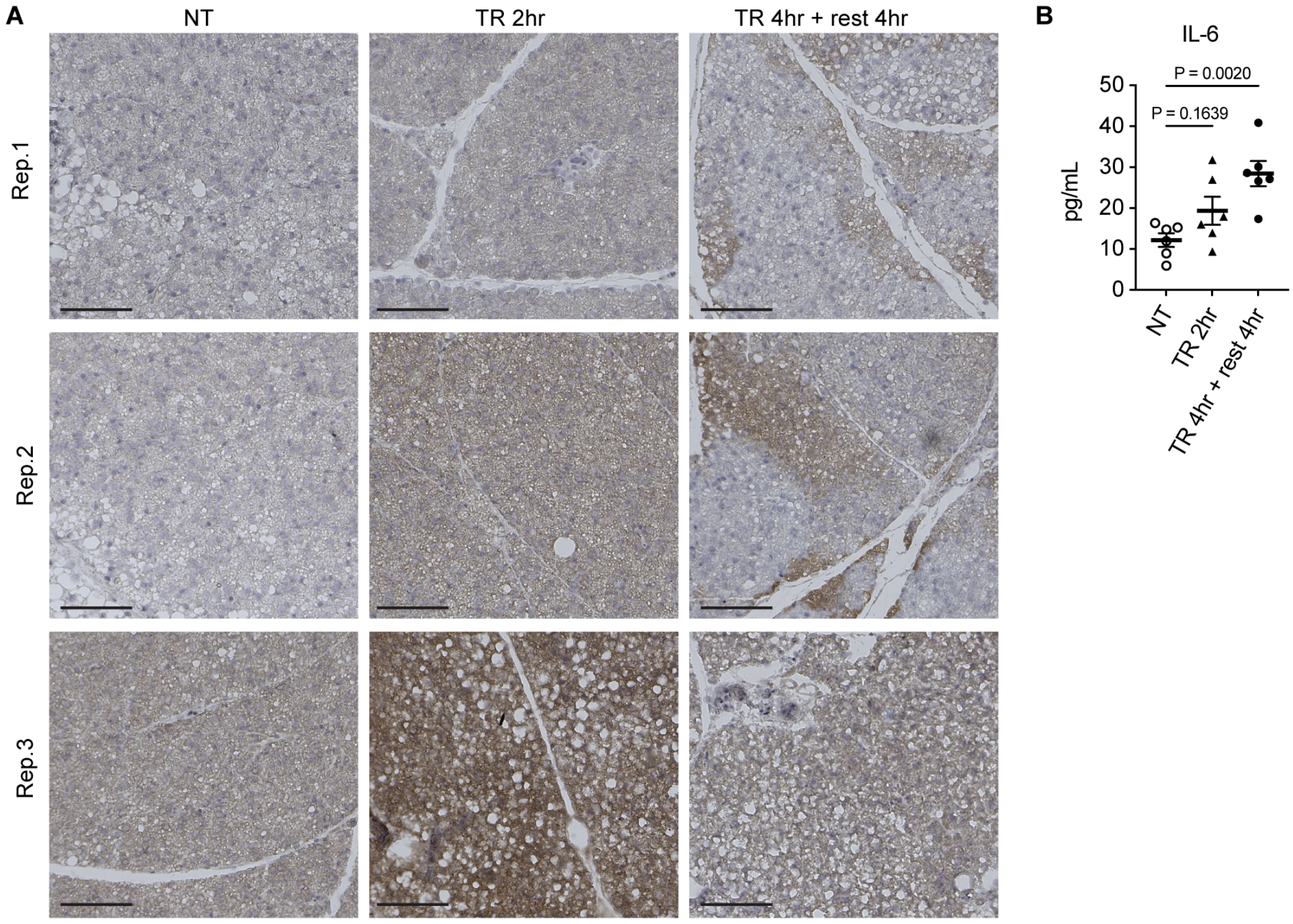

fig. S5. Enhanced IL-6 production in BAT followed by the restraint stress. (A) Representative images of BAT from untreated mice, mice subjected to 2 h of restraint stress, and mice subjected to 4 h of restraint stress followed by 4 h of rest. Images are representative of three independent samples. Scale bars, 100 µm. **(B)** Serum IL-6 levels following tube restraint stress (TR). N = 6 mice from 2 independent experiments. P values were determined by one-way ANOVA followed by Tukey's multiple-comparisons test.

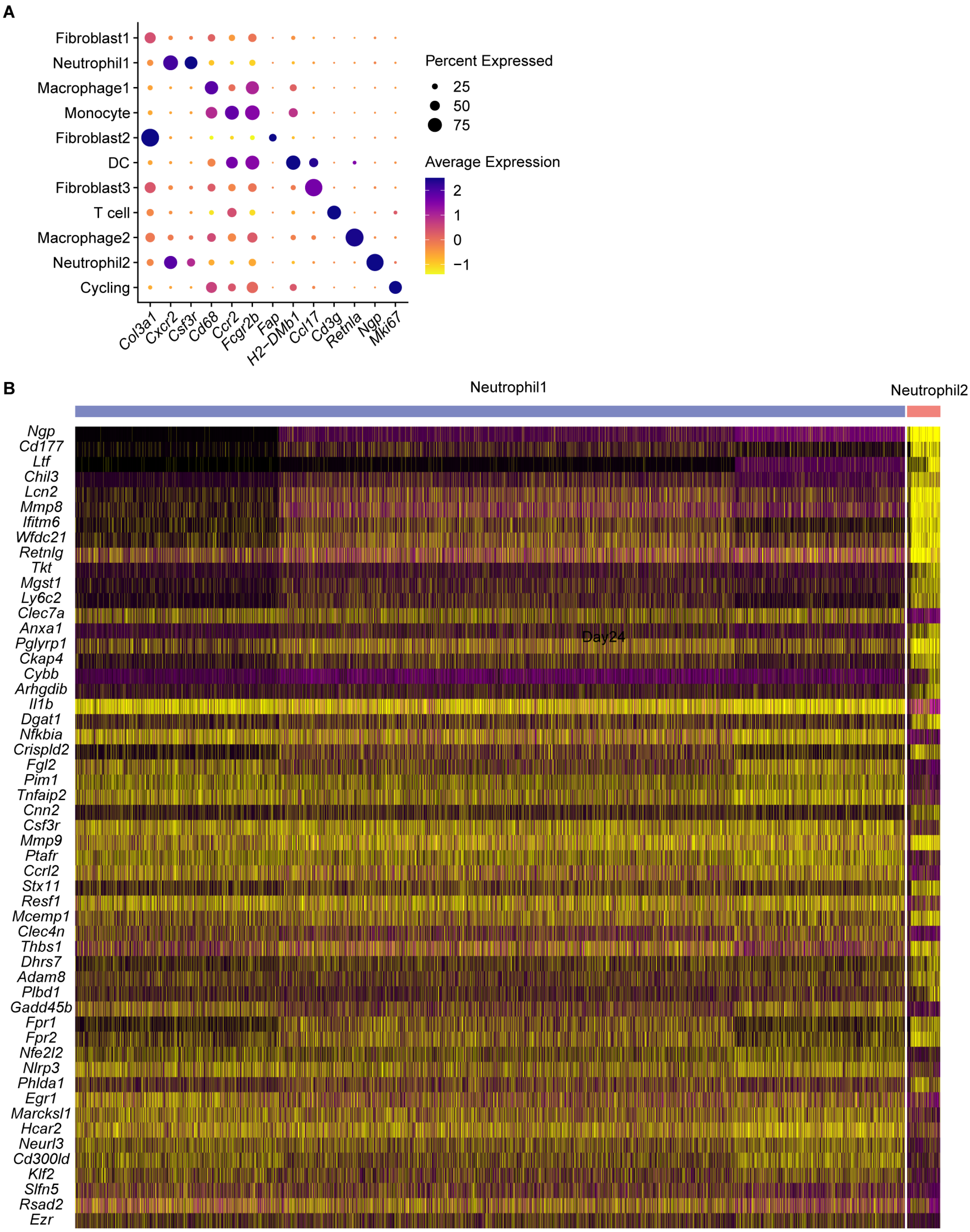

fig. S6. Characteristic gene expressions in single-cell RNA-sequencing clusters. (**A**) A bubble plot indicating characteristic gene expression within each cluster. (**B**) Heatmap displaying differentially expressed genes between Neutrophil1 and Neutrophil2 clusters.

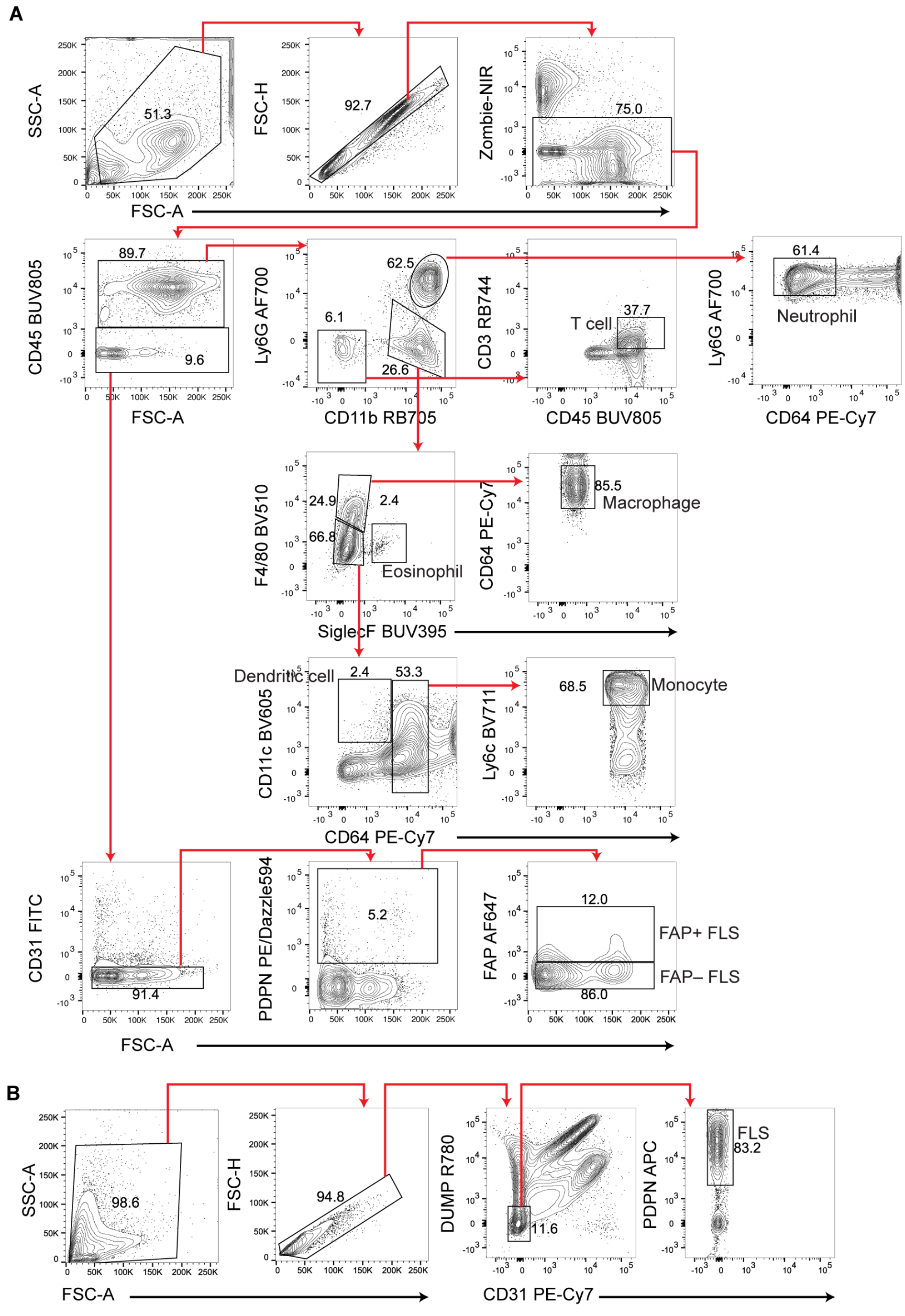

fig. S7. The gating strategy for Fig. 4E and 4G. (**A**) The gating strategy used to identify various cell types infiltrating the murine synovium of the knee joints is depicted. (**B**) The gating strategy employed to identify FLS in human synovial samples is illustrated.

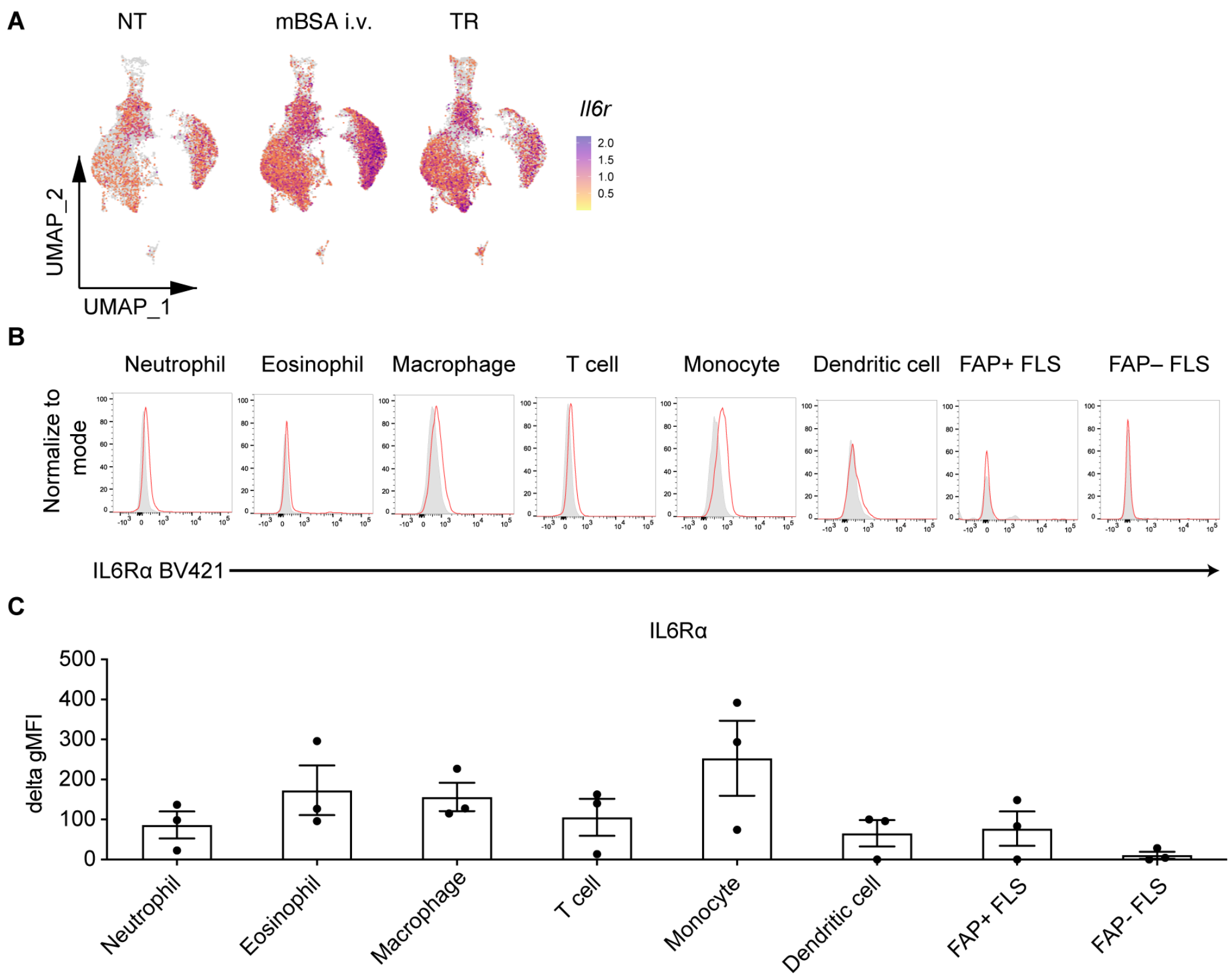

fig. S8. IL-6Rα is not expressed on macrophages and FAP+ FLS in synovium. (**A**) The expression of *Il6r* projected onto the UMAP plot. (**B**) Representative FACS plots of IL-6Rα in each synovial cell type during the remission state. (**C**) Cumulative data of IL-6Rα expression. The values represent the geometric mean fluorescence intensity (gMFI) of each sample, normalized by subtracting the isotype control. N = 3 from 2 independent experiments.

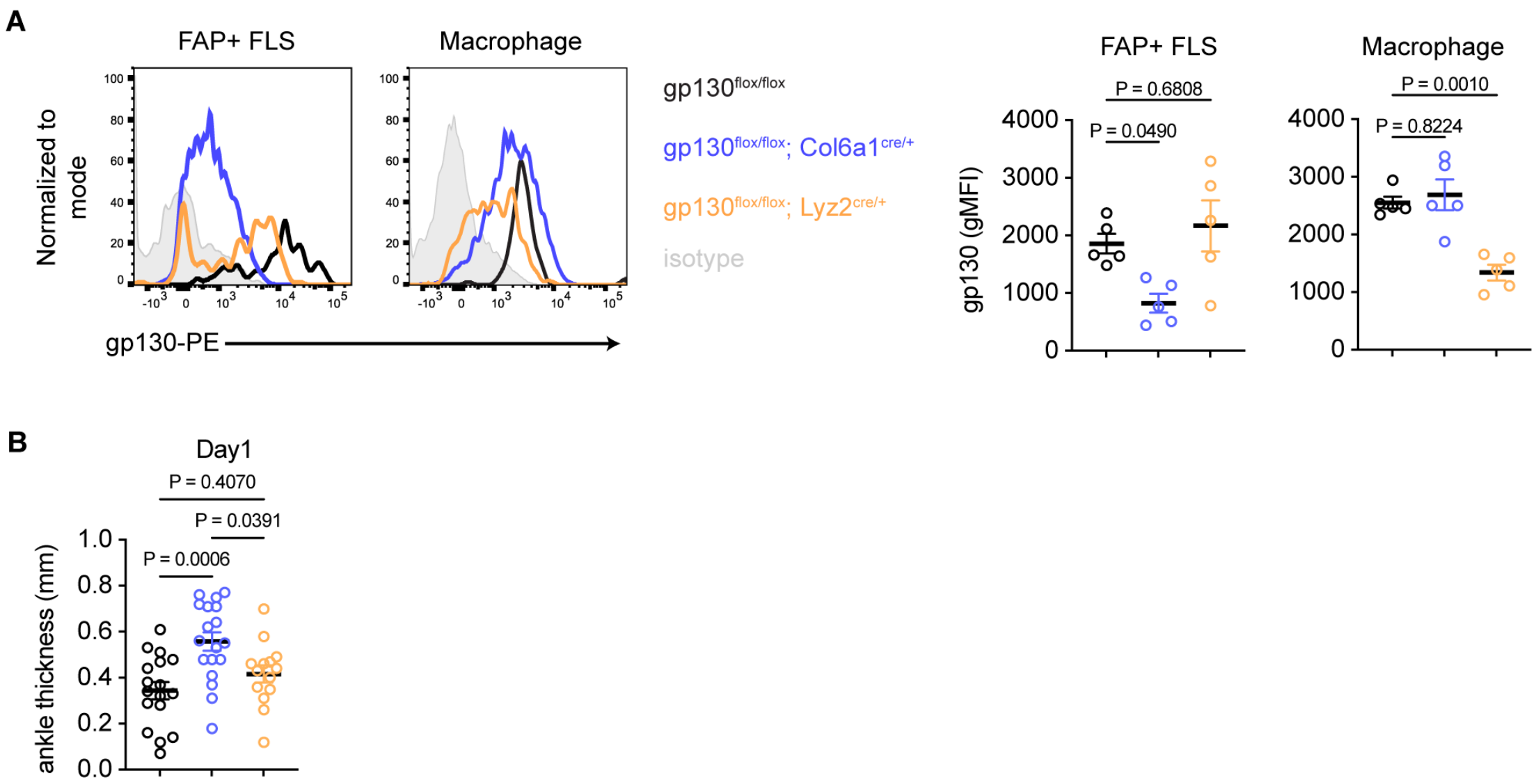

fig. S9. Supplementary data for Figure 4I to 4M (A). Confirmation of gp130 ablation in conditional gp130 knockout mice. Representative FACS plots of gp130 expression in FAP+ FLS and macrophages are shown. (**B**). Ankle thickness was measured at Day 1. N = 14 – 18 from 5 independent experiments. Statistical analyses were done by One-way ANOVA test followed by Tukey test.

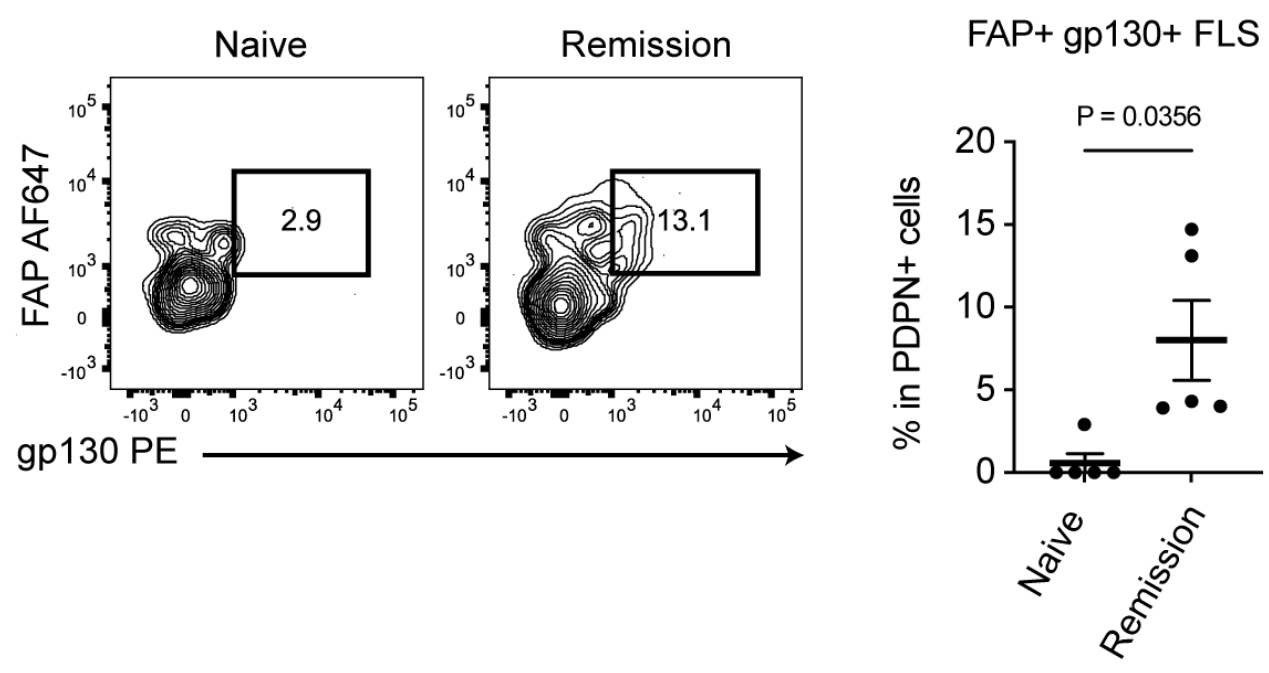

fig. S10. FAP+ gp130+ fibroblast-like synoviocytes in naïve and mice in remission state. Synovial cells isolated from knee joints of naïve mice and mice in remission were analyzed by flow cytometry. N = 5 mice from 2 independent experiments. P values were determined by an unpaired t test.

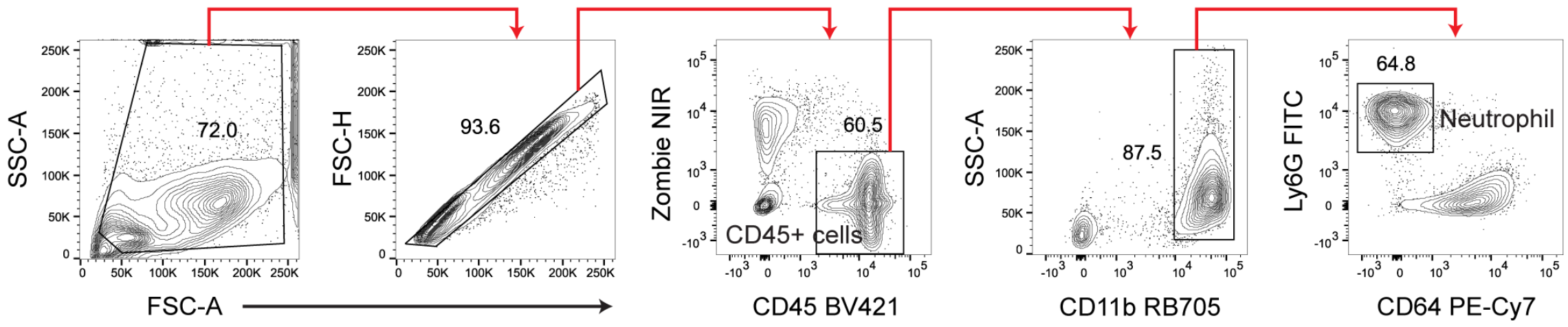

fig. S11. The gating strategy for identifying synovial neutrophils. The gating strategy for identifying synovial neutrophils was illustrated.

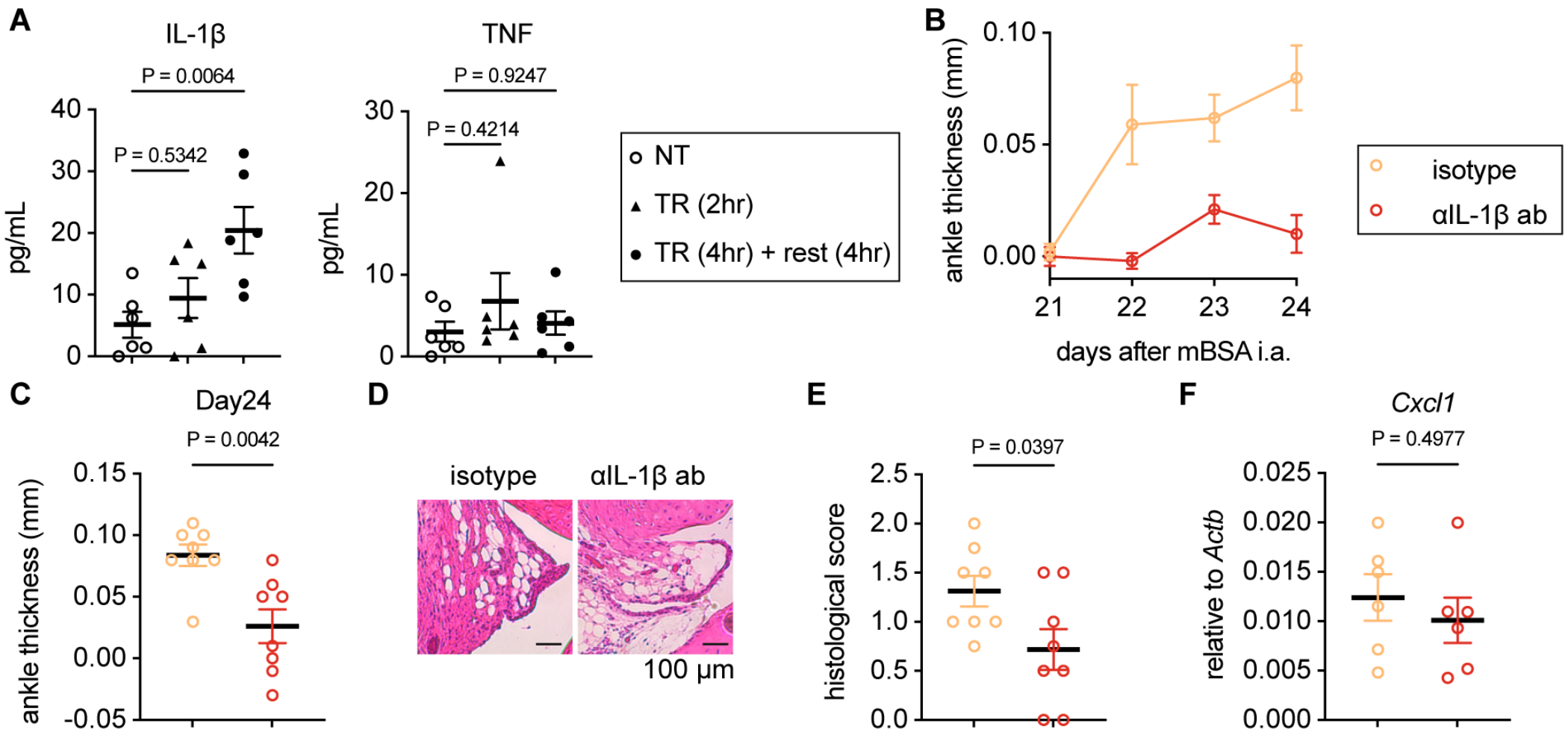

fig. S12. IL-1β as an amplifier of psychological stress-induced arthritis flare. (A) Serum levels of IL-1β and TNF were determined. P values were determined by one-way ANOVA followed by Tukey's multiple-comparisons test. **(B-F)** Three weeks after intra-articular mBSA injection, mice were subjected to tube restraint stress. During this procedure, mice were administered either an isotype control antibody or an anti-IL-1β antibody. **(B)** Ankle thickness after the intervention. **(C)** Ankle thickness at Day24. **(D)** Representative histological images. **(E)** Histological scores. **(B - E)** N = 8 from 3 independent experiments. **(F)** Cxcl1 mRNA levels in affected synovial tissues. N = 6 mice from 2 independent experiments. P values in B–F were determined by an unpaired t test.

Table S1. Patient characteristics for Fig. 4G and 4H

Characteristic	OA<br>N = 10	RA<br>N = 10	p-value[1]
age, Median (IQR)	71 (65 – 73)	69 (65 – 76)	0.79
duration, Median (IQR)	NA	10 (5 – 18)	
sex, Female, n (%)	9 (90)	10 (100)	>0.99
surgical site, n (%)			0.37
Hip	6 (60)	9 (90)	
Knee	2 (20)	1 (10)	
Sholder	2 (20)	0 (0)	
CCP positivity, n (%)	NA	6 (60)	

[1]Wilcoxon rank sum test; Fisher's exact test, OA; osteoarthritis, RA; rheumatoid arthritis, CCP; anti cyclic citrullinated peptide antibody, IQR; interquartile range, NA; not applicable.

Table S2. Patient characteristics for Fig. 5F

Characteristic	N = 9
age, Median (IQR)	73 (69 – 76)
duration, Median (IQR)	16 (10 – 23)
sex, Female, n (%)	9 (100)
surgical site, n (%)	
Hip	5 (56)
Knee	4 (44)
CCP positivity, n (%)	7 (78)

IQR; interquartile range, CCP; anti cyclic citrullinated peptide antibody

Table S3. Patient characteristics for Fig. 7

**Characteristic**	**Wild**<br>N = 127	**Mutant**<br>N = 61	**p-value**[1]
age, Median (IQR)	67 (56 – 73)	67 (56 – 75)	0.95
duration, Median (IQR)	10 (3 – 18)	10 (2 – 18)	0.83
sex, Female, n (%)	104 (82)	50 (82)	0.99
MTX, Median (mg, IQR)	6.0 (0.0 – 10.0)	0.0 (0.0 – 8.0)	0.010
PSL, Median (mg, IQR)	0.00 (0.00 – 2.00)	0.00 (0.00 – 0.00)	0.058
b/tsDMARDs use, n (%)	61 (48)	41 (67)	0.013
CRP, Median (mg/dL, IQR)	0.12 (0.05 – 0.36)	0.08 (0.03 – 0.26)	0.11
CCP positivity, n (%)	98 (83)	48 (87)	0.48
CDAI, Median (IQR)	6.0 (2.5 – 11.0)	5.7 (1.8 – 10.0)	0.39
PSS, Median (IQR)	19 (15 – 23)	19 (16 – 22)	>0.99

IQR; interquartile range, MTX; methotrexate, PSL; prednisolone, b/tsDMARDs; biologic/targeted synthetic disease modifying drugs, CRP; C-reactive protein, CCP; anti-cyclic citrullinated peptide antibody; CDAI; clinical disease activity index, PSS; perceived stress scale.

[1]Wilcoxon rank sum test; Pearson's Chi-squared test

Table S4. Antibodies used for flow cytometry

Antigen	Fluorescence	Clone	Company	Cat number	dilution
Siglec-F	BUV395	E50-2440	BD	740280	1:100
CD90.2	BUV737	30-H12	BD	741702	1:100
CD45	BUV805	30-F11	BD	568336	1:200
CD126	BV421	D7715A7	BD	740038	1:100
F4/80	BV510	BM8	BioLegend	123135	1:100
CD11c	BV605	N418	BD	744179	1:200
Ly-6C	BV711	HK1.4.rMAb	BD	755195	1:100
CD31	FITC	390	BioLegend	102406	1:100
CD11b	RB705	M1/70	BD	570292	1:100
CD3	RB744	17A2	BD	570561	1:200
CD130	PE	4H1B35	BioLegend	149404	1:100
PDPN	PE/Dazzle 594	8.1.1	BioLegend	127420	1:100
CD64	PE/Cy7	W18349C	BioLegend	164414	1:100
FAP	AF647	983802	R&D systems	FAB9727R	1:100
Ly-6G	AF700	1A8	BioLegend	127622	1:100
CD4	BV421	RM4-5	BioLegend	100563	1:200
CD8a	PE	53-6.7	BioLegend	100708	1:200
CD4	BUV615	H129.19	BD	751434	1:200
CD19	PE	6D5	BioLegend	115508	1:100
IFNg	BV510	XMG1.2	BioLegend	505842	1:100
IL-17A	PE	TC11-18H10.1	BioLegend	506904	1:100
CD3	PE-Cy7	17A2	BioLegend	100220	1:200
CD45	BV421	30-F11	BD	563890	1:100
Ly6G	FITC	1A8	BioLegend	127606	1:200
hCD31	PE-Cy7	WM59	BD	563651	1:500
hPDPN	APC	NZ-1.3	eBioscience	17-9381-42	1:100
hCD45	APC-H7	2D1	BD	641408	1:300
hCD130	RB705	AM64	BD	756844	1:100

Data File S1. Data spreadsheet. These spreadsheets contain data used to produce Figure 1-7 and S1-5, 8-10, and 12.
